# Rho GTPase–driven stress granules enable anchorage-independent survival under adhesion stress

**DOI:** 10.21203/rs.3.rs-10284597/v1

**Published:** 2026-10-01

**Authors:** Seungwon Yang, Anaïs Aulas, Paul J. Anderson, Pavel Ivanov

**Affiliations:** 1 Division of Rheumatology, Inflammation, and Immunity, Department of Medicine, Brigham and Women’s Hospital, Harvard Medical School, Boston, MA 02115, USA.

**Keywords:** Stress granules, adhesion stress, FAK, Rho GTPase, ROCK, PERK, eIF2α, anoikis resistance, anchorage-independent survival, cancer adaptation

## Abstract

Cells that lose adhesion to their native extracellular matrix must either adapt to impaired adhesion signaling or undergo anoikis. How cells convert loss of adhesion into translational stress responses and survival signaling remains incompletely understood. Here, we define adhesion stress as a cellular stress state triggered by impaired adhesion signaling and show that this state induces stress granule (SG) assembly as an adaptive survival mechanism. We modeled adhesion stress through cell detachment, reduced adhesion strength, and pharmacologic inhibition of focal adhesion kinase (FAK). Across these models, adhesion stress activated a RhoA–ROCK–PERK–eIF2α signaling pathway that drove SG assembly. PERK was the principal eIF2α kinase required for this adhesion-stress response. Proteomic profiling further showed that adhesion-stress SGs represent a previously unrecognized SG subtype characterized by a Rho GTPase-associated protein signature and selective enrichment of CASP4, an inflammatory caspase linked to cell-death signaling. Functionally, SG assembly limited CASP4 activation and reduced apoptotic signaling during FAK inhibitor-induced adhesion stress. In parallel, PERK contributed to sustained AKT–mTOR survival signaling during anoikis. Genetic loss of SG assembly or pharmacologic inhibition of G3BP-dependent SG formation increased anoikis sensitivity and sensitized cancer cells to multiple FAK inhibitors. SGs were also enriched in invading cancer cells in human breast tumors, consistent with a role for adhesion-stress adaptation in invasive tumor contexts. Together, these findings identify adhesion-stress SGs as a RhoA–ROCK–PERK-driven survival program that limits anoikis and enables anchorage-independent cancer cell survival.

## INTRODUCTION

Cell adhesion is a central organizer of cell signaling, cytoskeletal architecture, and survival. Engagement of the extracellular matrix through integrins and focal adhesion complexes supports cellular homeostasis by coordinating mechanical inputs with intracellular signaling pathways. Conversely, loss or weakening of adhesion disrupts focal adhesion signaling, alters actomyosin organization, and can trigger anoikis, a form of programmed cell death that prevents inappropriate survival outside the proper tissue context [1–4]. How cells sense impaired adhesion as a stress condition and convert this information into adaptive changes in translation and cell fate remains incompletely understood.

Stress granules (SGs) are non-membranous cytoplasmic ribonucleoprotein condensates that assemble when translation initiation is suppressed[5–8]. Canonical SG assembly is commonly initiated by phosphorylation of eIF2α through one of four stress-responsive eIF2α kinases: HRI, PKR, PERK, or GCN2[9]. Phosphorylated eIF2α limits ternary complex availability, reduces translation initiation, and promotes accumulation of stalled initiation complexes that nucleate SG assembly[5–8]. SGs can also form through eIF2α-independent mechanisms, including inhibition of cap-dependent eIF4F translation initiation downstream of mTORC1 suppression and 4E-BP1 activation[10–13]. Through these mechanisms, SGs allow cells to rapidly remodel translation and RNA metabolism in response to stress.

SGs were initially described as a general feature of stressed cells, but accumulating evidence indicates that their composition, assembly mechanism, and biological function differ according to the inducing stress and cellular context[7, 14–16]. SGs can limit stress-induced apoptosis, reduce reactive oxygen species, and modulate stress-responsive signaling pathways[17–19]. These observations support the view that SGs are not simply passive aggregates of translationally stalled mRNPs, but regulated stress-adaptive assemblies that influence cell fate. However, whether loss of adhesion can itself act as a stress signal that induces SG assembly has not been clearly defined.

Several adhesion-linked signaling pathways are positioned to connect impaired adhesion to translational stress responses. Focal adhesion kinase (FAK) integrates integrin-dependent adhesion cues with cytoskeletal and survival signaling[20–24]. AKT–mTOR signaling, which is regulated by adhesion and growth factor inputs, controls translation initiation through mTORC1 and 4E-BP1[12, 25, 26]. Rho GTPases and ROCK regulate actomyosin contractility, cell shape, and responses to altered adhesion states, and RhoA–ROCK signaling has also been implicated in SG formation[27–31]. PERK, an eIF2α kinase with established roles in cellular adaptation and tumor progression, can function downstream of ROCK signaling[32–34]. These observations raise the possibility that impaired adhesion activates a defined cell-biological stress pathway linking RhoA–ROCK signaling to PERK-dependent translational control and SG assembly.

This question is particularly relevant to cancer cells, which must survive altered adhesion states during invasion, dissemination, and anchorage-independent growth[2, 3, 35, 36]. Cancer cells frequently maintain survival signaling despite weakened adhesion, yet the stress-adaptive mechanisms that operate between loss of adhesion and execution or avoidance of anoikis remain poorly understood. We refer to this intermediate condition as adhesion stress: a cellular stress state caused by impaired adhesion signaling, including physical detachment, reduced adhesion strength, and pharmacologic inhibition of FAK.

Here, we show that impaired adhesion activates a RhoA–ROCK–PERK–eIF2α pathway that induces SG assembly during adhesion stress and supports anchorage-independent survival. Proteomic analysis identifies adhesion-stress SGs as a previously unrecognized Rho GTPase-associated SG subtype enriched for CASP4[37–39], linking SG composition to cell death regulation. We further show that PERK sustains AKT–mTOR survival signaling during anoikis, whereas genetic or pharmacologic disruption of G3BP-dependent SG assembly increases anoikis sensitivity and sensitizes cancer cells to FAK inhibitors. These findings place SG assembly within the cell-biological response to impaired adhesion and reveal a stress-response mechanism that can be exploited in adhesion-stress-adapted cancer cells.

### 1. Loss of adhesion engages an eIF2α stress response and induces stress granule assembly

To determine whether loss of adhesion itself functions as a cellular stress signal, we examined activation of the eIF2α stress pathway during EDTA-mediated cell detachment. EDTA was used to induce detachment without proteolytic cleavage of cell-surface receptors, as occurs with trypsin. Acute EDTA treatment induced rapid cell rounding and detachment across multiple cell types (Fig. 1A). This was accompanied by increased eIF2α phosphorylation across U2OS, MEF, HeLa, MDA-MB-468, MCF10A, MDA-MB-361, SKBr3, MCF7, and MDA-MB-231 cells (Fig. 1B–G). In parallel, detachment reduced phosphorylation of AKT and the mTORC1 downstream target 4E-BP1 and decreased phosphorylation of adhesion-associated proteins, including FAK and paxillin, in the cell lines analyzed for these markers (Fig. 1B–G). Although the magnitude and pattern of these signaling changes varied among cell types, these findings indicate that impaired adhesion is sufficient to activate an eIF2α-linked stress response while attenuating AKT–mTOR and focal adhesion signaling.

We then tested whether this response was reversible and dependent on adhesion strength. Replating detached cells onto fibronectin-coated substrates promoted cell spreading, paxillin phosphorylation, and actin organization, while eIF2α phosphorylation progressively declined (Fig. 1H,I). Likewise, increasing fibronectin concentration enhanced adhesion signaling, as shown by increased phosphorylation of FAK and paxillin, whereas PYK2 phosphorylation remained largely unchanged, and was accompanied by reduced eIF2α phosphorylation as adhesion strength increased (Fig. 1J,K). These data show that eIF2α phosphorylation is dynamically and inversely regulated by adhesion strength while suppressing adhesion-linked survival and focal adhesion signaling.

We next asked whether this adhesion-regulated stress response leads to stress granule assembly. EDTA-mediated detachment rapidly induced G3BP1-positive granules during live-cell imaging (Fig. 1L and Supplementary Video A). We further examined SG formation in two fixed-cell conditions: during early re-adhesion after replating onto fibronectin, before full cell spreading (Fig. 1M), and during acute EDTA-induced detachment (Fig. 1N). G3BP1- and TIA-1-positive granules were observed during early re-adhesion, whereas G3BP1-positive granules were detected during EDTA-induced detachment (Fig. 1M, N). These results establish adhesion stress as a cellular stress state marked by eIF2α phosphorylation and SG assembly.

### 2. Pharmacologic FAK inhibition engages adhesion-stress signaling and induces canonical stress granule assembly

We next asked whether pharmacologic inhibition of focal adhesion signaling is sufficient to reproduce the stress response observed during loss of adhesion. We therefore treated cells with FAK14, a small-molecule FAK inhibitor, and examined adhesion-linked signaling together with eIF2α phosphorylation. In U2OS cells, FAK14 reduced phosphorylation of FAK and paxillin in a dose-dependent manner and was accompanied by increased eIF2α phosphorylation and reduced phosphorylation of the mTORC1 downstream target 4E-BP1 (Fig. 2A). Similar signaling changes were observed in a panel of breast cancer cell lines, including SKBr3, MCF7, MDA-MB-361, and MDA-MB-231 cells, although the magnitude of the response varied among cell types (Fig. 2B–E). In contrast, PYK2 phosphorylation was not increased under these acute treatment conditions (Fig. 2A), suggesting that this pharmacologic model suppresses FAK-linked adhesion signaling without a clear compensatory increase in PYK2. These findings indicate that inhibition of FAK signaling is sufficient to engage an eIF2α-linked stress response while attenuating focal adhesion and AKT–mTOR-associated signaling.

We then examined whether FAK inhibition induces SG assembly. FAK14 treatment promoted the formation of G3BP1-positive cytoplasmic granules that also contained the SG-associated proteins eIF4G and FXR1 (Fig. 2F, G). SG formation increased with FAK14 dose and exposure time, with robust granule assembly observed after higher-dose acute treatment (Fig. 2F–H and Supplementary Fig. S1A). To determine whether this response was specific to FAK14 or reflected a broader consequence of FAK inhibition, we tested two additional FAK inhibitors, PF-562271 and defactinib. In contrast to FAK14, these inhibitors produced more delayed morphological changes and did not rapidly induce cell rounding or detachment within the first few hours. Nevertheless, prolonged treatment with either PF-562271 or defactinib induced SGs containing G3BP1, eIF4G, and FXR1, with marked granule accumulation by 48 h (Fig. 2I–K and Supplementary Fig. S1B). Low-dose FAK14 treatment for extended periods also induced SG formation, supporting the view that sustained attenuation of FAK signaling is sufficient to promote this response (Supplementary Fig. S1C).

We next assessed whether FAK inhibitor-induced granules displayed features of canonical SGs. FAK14-induced granules contained G3BP1 together with eIF3B and TIA-1, similar to sodium arsenite-induced canonical SGs[40](Fig. 2L). These findings indicate that FAK inhibition induces SG assembly with a canonical SG marker profile. Consistent with the morphological effects of impaired adhesion signaling, live-cell imaging further confirmed that FAK14 induces SG assembly in living cells. In G3BP1/2-deficient cells reconstituted with EGFP-G3BP1, FAK14 treatment induced EGFP-G3BP1-positive granules as cells developed a contracted morphology and underwent detachment (Fig. 2M and Supplementary Video B). Thus, pharmacologic FAK inhibition phenocopies key features of adhesion stress by linking suppression of focal adhesion signaling to eIF2α phosphorylation and canonical SG assembly.

### 3. Adhesion stress induces SG assembly primarily through a ROCK–PERK–eIF2α pathway

We next sought to define the signaling mechanism linking impaired adhesion signaling to SG assembly. Because adhesion stress increased eIF2α phosphorylation, we first examined which eIF2α kinase was required for this response. Using MDA-MB-468 cells deficient in GCN2, PKR, HRI, or PERK, we compared signaling responses in adherent and suspension conditions. Among these kinase-deficient cells, loss of PERK most strongly impaired eIF2α phosphorylation during suspension, whereas loss of GCN2, PKR, or HRI did not produce a comparable effect (Fig. 3A and Supplementary Fig. S2A, B). PERK-deficient cells also failed to suppress global protein synthesis in response to suspension, as assessed by puromycin incorporation (Fig. 3B). Consistent with these signaling defects, PERK loss markedly reduced FAK inhibitor-induced SG assembly, although residual SG formation remained (Fig. 3C, D). These findings identify PERK as the principal eIF2α kinase and a major driver of SG assembly during adhesion stress, while suggesting that additional inputs may contribute to the full SG response.

We then asked how impaired adhesion signaling activates PERK. PERK has been reported to function downstream of ROCK signaling[32], and RhoA–ROCK signaling has also been linked to SG formation and apoptosis[27, 28, 41]. We therefore examined whether this ROCK–PERK signaling relationship operates during adhesion stress. RhoA activity was increased in suspension cells compared with adherent or fibronectin-replated cells (Fig. 3E). Pharmacologic inhibition of ROCK with Y-27632 reduced eIF2α phosphorylation under both EDTA-induced suspension and FAK inhibitor conditions, while also suppressing phosphorylation of the ROCK substrate MLC2 (Fig. 3F, H). ROCK inhibition reduced FAK14-induced PERK and eIF2α phosphorylation without restoring FAK or paxillin phosphorylation (Fig. 3G, H). These results suggest that ROCK contributes to PERK–eIF2α activation during adhesion stress.

Consistent with this signaling relationship, ROCK inhibition markedly reduced the percentage of SG-positive cells after FAK14 treatment (Fig. 3I, J).

Because residual SG formation remained after PERK loss, and because FAK inhibition also reduced 4E-BP1 phosphorylation, we considered whether attenuation of the mTORC1–eIF4F axis contributes to SG assembly in parallel with PERK–eIF2α signaling[42]. Cap-binding assays using m^7^GTP-Sepharose showed that FAK14 increased the association of 4E-BP1 with cap-bound eIF4E and reduced recovery of eIF4F components, including eIF4G and eIF4A, although this effect was weaker than that induced by mTOR inhibition (Fig. 3K). We therefore tested whether 4E-BP1 was functionally required for this component of the response. In 4E-BP1-deficient U2OS cells, FAK14-induced SG assembly was modestly reduced, particularly at higher FAK14 concentration, but was not abolished (Fig. 3L–N and Supplementary Fig. S2C). These findings suggest that 4E-BP1-dependent disruption of eIF4F contributes to FAK inhibitor-induced SG assembly but represents a secondary input rather than the dominant mechanism.

We finally tested the requirement for eIF2α phosphorylation directly using Hap1 cells expressing a non-phosphorylatable eIF2α-S51A mutant. As expected, eIF2α-S51A cells failed to increase eIF2α phosphorylation after sodium arsenite treatment (Fig. 3O). FAK14-induced SG assembly was completely suppressed in eIF2α-S51A cells, indicating that phosphorylation of eIF2α at Ser51 is required for this response (Fig. 3P, Q and Supplementary Fig. S2D). In contrast, ROCK inhibition did not block sodium arsenite-induced SG formation, supporting the specificity of ROCK signaling for adhesion-stress conditions rather than SG assembly in general (Fig. 3R, S and Supplementary Fig. S2E). In MDA-MB-468 cells, ROCK inhibition also strongly reduced FAK14-induced SG assembly, consistent with a prominent contribution of the ROCK–PERK–eIF2α axis in this breast cancer model (Fig. 3T, U and Supplementary Fig. S2F). These data support a signaling hierarchy in which adhesion stress activates ROCK and PERK to drive eIF2α-dependent SG assembly, with a secondary contribution from 4E-BP1-dependent disruption of eIF4F.

### 4. Proteomic analysis identifies a Rho GTPase-associated adhesion-stress SG state linked to CASP4 regulation

Because SG composition can vary according to the inducing stress, we next asked whether SGs formed during adhesion stress possess a distinct protein composition. To distinguish SG-dependent recruitment from nonspecific binding to GFP-tagged proteins, we expressed EGFP-empty, EGFP-G3BP1, or the SG-defective EGFP-G3BP1-ΔNTF2 mutant in G3BP1/2-deficient cells and isolated G3BP1-associated complexes after treatment with FAK14 or sodium arsenite (SA) (Fig. 4A). GFP-Trap samples were validated by immunoblotting for established SG-associated proteins, including eIF4G, USP10, and FXR1, which were enriched in EGFP-G3BP1 pull-downs under SG-forming conditions compared with EGFP-empty and ΔNTF2 controls (Fig. 4B, C). These samples were then analyzed by mass spectrometry.

Proteomic analysis identified both shared and stress-selective SG-associated proteins. After filtering against EGFP-empty controls and the SG-defective EGFP-G3BP1-ΔNTF2 mutant, 254 proteins were associated with FAK14-induced SGs and 316 proteins were associated with SA-induced SGs. Among these proteins, 127 proteins were shared between the two SG conditions, whereas 127 proteins were selectively associated with FAK14-induced SGs and 189 proteins were selectively associated with SA-induced SGs (Fig. 4D–G). Gene Ontology and pathway enrichment analysis showed that FAK14-selective SG proteins were enriched for several functional categories, including Rho GTPase-related signaling (Fig. 4F and Supplementary Fig. S3A, B). Reactome-based pathway annotation further showed that proteins assigned to the “Signaling by Rho GTPases” pathway were represented in the FAK14-selective and shared SG fractions, but not in the SA-selective fraction (Fig. 4G). These findings indicate that adhesion-stress SGs are compositionally distinct from SA-induced SGs and are enriched for proteins linked to Rho GTPase-associated signaling.

We next validated representative proteins from the FAK14-associated SG proteome. XPO1, a candidate from the Rho GTPase-associated cluster, was enriched in EGFP-G3BP1 pull-downs after FAK14 treatment and was also detected in a subset of FAK14-induced SGs by immunofluorescence (Fig. 4H–J). These results support the proteomic identification of Rho GTPase-associated proteins as components of adhesion-stress SGs.

The proteomic dataset also identified CASP4 as a cell death-associated protein selectively enriched in FAK14-induced SGs (Fig. 4E, G). Immunoblotting of GFP-Trap samples confirmed that full-length CASP4, rather than cleaved CASP4, was associated with EGFP-G3BP1 complexes after FAK14 treatment (Fig. 4H). Immunofluorescence further showed that a subset of FAK14-induced SGs contained CASP4, as detected together with G3BP1 and eIF4G (Fig. 4K and Supplementary Fig. S3C, D). These findings suggest that CASP4 is recruited to adhesion-stress SGs predominantly in an uncleaved form.

We then asked whether SG assembly affects CASP4 activation during adhesion stress. FAK14 treatment increased CASP4 activity in G3BP1/2-deficient cells compared with control cells, indicating that loss of SG assembly permits enhanced CASP4 activation under FAK inhibitor-induced adhesion stress (Fig. 4L). In MDA-MB-468 cells, CASP4 knockdown partially rescued the survival defect of G3BP1/2-deficient cells under anchorage-independent conditions (Fig. 4M, N). These findings identify adhesion-stress SGs as a compositionally distinct, Rho GTPase-associated SG subtype that includes CASP4 and limits CASP4 activation during impaired adhesion signaling.

### 5. PERK sustains AKT–mTOR survival signaling during anoikis, whereas SG assembly remains partly independent of growth pathway activity

Because PERK was required for adhesion-stress SG assembly, we next asked whether PERK also contributes to survival signaling in breast cancer cells exposed to adhesion stress. Analysis of TCGA breast invasive carcinoma datasets showed that EIF2AK3, which encodes PERK, was significantly elevated in primary breast tumors compared with normal breast tissue. Several representative PERK–ATF4 integrated stress response genes, including ASNS, TRIB3, SHMT2, SLC7A11, and CHAC1, were also increased in tumor samples, whereas SESN2 was not significantly changed and DDIT3 showed only a modest increase[43–46] (Fig. 5A). Consistent with this pattern, PERK protein was detected across a panel of breast cell lines, including ER-positive, HER2-positive, and triple-negative breast cancer models (Fig. 5B). These observations suggest that the PERK–ATF4 stress response program is elevated in human breast cancer and is retained across multiple breast cancer cell contexts.

We then examined whether PERK contributes to cellular fitness under adhesion stress. In MDA-MB-468 cells, loss of PERK reduced the CCK-8 viability signal under both adherent and suspension conditions (Fig. 5C), suggesting that PERK supports cellular fitness in both anchorage-dependent and anchorage-independent states. Although acute detachment initially reduced AKT signaling, MDA-MB-468 cells showed renewed AKT phosphorylation during prolonged adhesion-stress conditions. In line with this adaptive pattern, FAK14 treatment induced a gradual increase in AKT phosphorylation over time (Fig. 5D).

To examine this response under controlled detachment conditions, cells were detached with EDTA and maintained in suspension on poly(HEMA)-coated dishes (Fig. 5E). Because PTEN restrains PI3K–AKT signaling[27, 47, 48], we examined whether PTEN-deficient MDA-MB-468 cells maintain AKT–mTOR activity during adhesion stress. During suspension culture, AKT–mTOR pathway activity was reduced in non-transformed MCF10A cells, whereas MDA-MB-468 cells retained AKT phosphorylation and phosphorylation of the mTORC1 downstream targets 4E-BP1 and S6 (Fig. 5F and Supplementary Fig. S4A). In MDA-MB-468 cells, PERK loss reduced the maintenance of AKT and S6 phosphorylation during prolonged suspension (Fig. 5G). These results indicate that PERK contributes to sustained AKT–mTOR survival signaling during anoikis in PTEN-deficient breast cancer cells.

We next asked whether growth pathway activity is required for SG assembly under metabolic stress. Under basal culture conditions, several breast cancer cell lines showed elevated S6 phosphorylation relative to non-transformed MCF10A cells, consistent with persistent PI3K–mTOR pathway activity associated with oncogenic growth signaling, including PI3K pathway activation or PTEN loss in this panel (Supplementary Fig. S4A). We then tested whether this signaling was retained during nutrient deprivation using HBSS containing Ca^2+^ and Mg^2+^. Even after HBSS treatment, S6 phosphorylation remained relatively high in several breast cancer cell lines compared with MCF10A cells, suggesting that these cancer cells retain mTORC1-linked growth pathway activity during metabolic stress (Fig. 5H). Under the same HBSS conditions, sodium arsenite-induced SG formation was reduced in MCF10A cells but was largely maintained in MDA-MB-231 and MDA-MB-468 cells (Fig. 5I, J). These data suggest that the growth-signaling state of cancer cells may influence SG assembly under metabolic stress.

However, acute inhibition of individual growth pathways did not prevent SG assembly under these conditions. In MDA-MB-468 cells, inhibition of MEK, PI3K, or mTOR, either alone or in combination, did not substantially reduce HBSS-associated SG formation, as assessed by G3BP1 and eIF4G staining (Fig. 5K, L). These findings suggest that PERK supports AKT–mTOR signaling during anoikis, but that AKT–mTOR activity itself is not a simple requirement for SG formation in this model. Rather, PERK appears to contribute to two related but distinct responses to adhesion stress: eIF2α-dependent SG assembly and maintenance of AKT–mTOR survival signaling during anchorage-independent growth.

### 6. G3BP-dependent SG assembly supports anchorage-independent survival and cancer cell fitness

Because adhesion-stress SGs showed distinct composition and were linked to CASP4 regulation, we next examined whether SGs are present in invasive tumor contexts and whether SG assembly supports cancer cell fitness. Immunostaining of human breast cancer tissue sections containing normal ductal structures and invasive carcinoma regions showed few G3BP1- and TIA-1-positive puncta in normal ducts, whereas SG-positive pan-keratin-positive tumor cells were more frequently observed in invasive regions (Fig. 6A). Quantification showed that SG-positive tumor cells were increased in collectively invading carcinoma cells and were most abundant in singly invading cancer cells (Fig. 6B). These observations are consistent with enrichment of SGs in breast cancer cells within invasive tissue contexts, where adhesion cues are likely altered.

We then tested whether SG assembly contributes to anchorage-independent survival. Loss of G3BP1/2 reduced survival under poly(HEMA)-induced anoikis stress in both U2OS and MDA-MB-468 cells (Fig. 6C, D and Supplementary Fig. S5A, B). This survival defect was further supported by an independent live-cell viability assay using Calcein AM, in which wild-type cells showed greater resistance to FAK inhibitor-induced anoikis stress than G3BP1/2-deficient cells (Supplementary Fig. S5C). Because G3BP1 and G3BP2 can differ in their contribution to SG assembly, we compared SG formation in single- and double-G3BP-deficient cells. As expected, G3BP1/2-deficient cells failed to assemble sodium arsenite-induced SGs, whereas loss of G3BP2 alone had little effect. In contrast, loss of G3BP1 reduced SG assembly, including SG size, indicating a stronger requirement for G3BP1 in this system (Supplementary Fig. S5D–G). We therefore used wild-type G3BP1 and the SG-defective G3BP1-ΔNTF2 mutant for reconstitution experiments. Reconstitution with wild-type G3BP1 rescued survival under suspension conditions, whereas G3BP1-ΔNTF2 did not (Fig. 6E, F and Supplementary Fig. S5D). These findings indicate that the SG-assembly function of G3BP1, rather than G3BP1 expression alone, supports cell survival under anchorage-independent conditions.

Consistent with this interpretation, reconstitution with wild-type G3BP1 reduced apoptotic activation during FAK inhibitor-induced adhesion stress. In G3BP1/2-deficient cells expressing EGFP-empty, EGFP-G3BP1, or EGFP-G3BP1-ΔNTF2, FAK14 treatment induced active caspase-3/7 signals in EGFP-empty and ΔNTF2-expressing cells. In contrast, cells expressing wild-type EGFP-G3BP1 formed SGs and showed little or no active caspase-3/7 signal (Fig. 6G, H). Together, these results show that G3BP-dependent SG assembly restrains apoptotic activation during FAK inhibitor-induced adhesion stress.

We next examined whether G3BP proteins support broader cancer cell activities. G3BP1/2-deficient U2OS and MDA-MB-468 cells showed reduced proliferative capacity compared with wild-type controls (Fig. 6I and Supplementary Fig. S5H–K). Reconstitution with wild-type G3BP1 restored proliferative capacity more effectively than G3BP1-ΔNTF2 (Fig. 6J, K). G3BP-deficient cells were also more sensitive to metabolic stress induced by HBSS, including in U2OS, MEF, and MDA-MB-468 models (Fig. 6L–N and Supplementary Fig. S5L, M). These data indicate that G3BP proteins support cellular fitness across both adhesion-related and metabolic stress conditions.

Finally, we assessed whether SG assembly influences migratory behavior. Loss of G3BP1/2 reduced U2OS cell migration, and this phenotype was rescued by wild-type G3BP1 but not by G3BP1-ΔNTF2 in both U2OS and MDA-MB-468 cells (Fig. 6O–Q and Supplementary Fig. S5N–R). Analysis of cells lacking additional SG-associated proteins, including CSDE1, YBX proteins, UBAP2L, and FXR family proteins, further showed that several SG components contribute to proliferation, anchorage-independent survival, or migration, although the magnitude and pattern of these effects varied among individual factors. Notably, UBAP2L loss did not detectably impair migration (Fig. 6R–T and Supplementary Fig. S5S). These findings indicate that G3BP-dependent SG assembly and associated SG components support multiple aspects of cancer cell fitness, including proliferation, anchorage-independent survival, metabolic stress tolerance, and migration. They also show that SG assembly limits apoptotic activation during FAK inhibitor-induced adhesion stress.

### 7. Pharmacologic inhibition of SG assembly sensitizes cancer cells to FAK inhibition

Because genetic disruption of G3BP-dependent SG assembly reduced survival under adhesion stress, we tested whether pharmacologic inhibition of SG formation could enhance the response to FAK inhibition. We used FAZ-3532, a small-molecule inhibitor of G3BP-dependent SG assembly, to disrupt SG formation. In MDA-MB-468 cells, FAZ-3532 reduced SG assembly induced by sodium arsenite or FAK14, as assessed by G3BP1 and eIF4G staining (Fig. 7A, B). Although FAZ-3532 did not uniformly eliminate all SG-positive cells, it markedly reduced the number of G3BP1- and eIF4G-positive granules per cell under both stress conditions (Fig. 7C, D). Similar effects on SG number or size were observed in U2OS and HeLa cells, although the extent of inhibition varied among cell lines (Supplementary Fig. S6A–H). These findings indicate that pharmacologic inhibition of G3BP-dependent assembly attenuates SG formation across multiple stress conditions and cell contexts.

We then tested whether genetic loss of SG assembly increases sensitivity to FAK inhibition. In dose-response assays using the FAK inhibitor GSK2256098, G3BP1/2-deficient U2OS cells showed greater sensitivity to FAK inhibition than wild-type cells (Fig. 7E). A similar sensitization was observed in G3BP1/2-deficient MDA-MB-468 cells, where loss of G3BP1/2 reduced the apparent resistance to GSK2256098 compared with wild-type controls (Fig. 7F). These results are consistent with the anoikis-survival data and support the conclusion that SG assembly limits the growth-inhibitory effects of FAK blockade.

To determine whether pharmacologic SG inhibition produces a similar effect, we combined FAZ-3532 with multiple FAK inhibitors across several cancer cell lines. FAZ-3532 enhanced the growth-inhibitory effects of FAK14, PF-562271, GSK2256098, or defactinib in multiple models, with clear sensitization observed in HeLa, MDA-MB-231, and MDA-MB-468 cells (Fig. 7G, J, K). The combination effect was more variable in MCF7 and SKBr3 cells, which showed relatively strong responses to some FAK inhibitors alone and less consistent additional suppression by FAZ-3532 (Fig. 7H, I). FAZ-3532 alone produced limited cytotoxicity under the conditions tested, supporting the interpretation that its major effect was to enhance vulnerability to FAK inhibitor-induced stress rather than to broadly reduce cell viability by itself (Supplementary Fig. S6I). Together, these findings indicate that pharmacologic inhibition of SG assembly can increase cancer cell sensitivity to FAK inhibitors in a context-dependent manner.

These data support a model in which loss of adhesion activates ROCK–PERK–eIF2α signaling to promote SG assembly. In parallel, PERK contributes to AKT–mTOR survival signaling during anoikis. Together, these responses support cancer cell survival under anchorage-independent conditions by limiting apoptotic and CASP4-associated cell death signaling. Genetic or pharmacologic disruption of G3BP-dependent SG assembly reduces this adaptive survival response and increases sensitivity to FAK inhibition (Fig. 7L). These findings suggest that SG assembly promotes anoikis resistance and may also be exploited to enhance FAK-directed therapies in adhesion-stress-adapted cancer cells.

## DISCUSSION

Our findings show that impaired adhesion activates a translational stress response before anoikis is fully executed. Across physical detachment, reduced adhesion strength, and pharmacologic FAK inhibition, impaired adhesion increased eIF2α phosphorylation and induced SG assembly. These findings extend the concept of SGs beyond conventional environmental stress responses and place SG formation within the early cellular response to altered adhesion. Thus, loss of adhesion is not only a disruption of survival signaling, but also a stress condition that engages translational remodeling.

Mechanistically, adhesion stress induced SG assembly primarily through a RhoA–ROCK–PERK–eIF2α pathway. Rho GTPase and ROCK signaling have established roles in actomyosin contractility, cell shape control, and altered adhesion states[27–31]. Our data place ROCK upstream of PERK–eIF2α activation during adhesion stress, providing a direct link between adhesion-regulated cytoskeletal signaling and the integrated stress response. Among the eIF2α kinases tested, PERK was the principal kinase required for eIF2α phosphorylation, translational inhibition, and efficient SG assembly. This mechanism suggests that RhoA–ROCK signaling can transmit adhesion stress to the translational machinery through PERK. FAK signaling has previously been implicated in SG dynamics during stress recovery, where FAK-dependent phosphorylation of Grb7 promotes SG disassembly[49]. Our findings extend this connection by showing that impaired FAK-linked adhesion signaling promotes SG assembly during adhesion stress through ROCK–PERK–eIF2α–activation.

Although PERK–eIF2α signaling was dominant, adhesion-stress SG assembly was not explained by a single linear pathway. Residual SG formation after PERK loss, together with the 4E-BP1 and cap-binding data, indicates that the mTORC1–4E-BP1–eIF4F axis contributes as a secondary input. This is consistent with prior work showing that SGs can form through eIF2α-independent mechanisms involving disruption of cap-dependent translation initiation[10–12]. At the same time, AKT–mTOR activity was not a simple upstream requirement for SG assembly in our model. Cancer cells with persistent mTORC1-linked growth signaling remained competent for SG formation under metabolic stress, and acute inhibition of MEK, PI3K, or mTOR did not prevent SG assembly in MDA-MB-468 cells. These results suggest that mTORC1–eIF4F regulation contributes to adhesion-stress SG formation, whereas broader growth signaling acts in a context-dependent or permissive manner.

Proteomic analysis separated adhesion-stress SGs from sodium arsenite-induced SGs at the level of protein composition. FAK14-induced SGs shared canonical SG components but were selectively enriched for Rho GTPase-associated proteins. This supports the emerging view that SG composition and function vary according to the inducing stress and cellular context[7, 14–16]. The enrichment of Rho GTPase-related proteins is notable because RhoA–ROCK signaling also acts upstream of SG assembly in this model. Adhesion-stress SGs may therefore arise from RhoA–ROCK signaling while also organizing proteins linked to altered adhesion and cytoskeletal remodeling.

CASP4 emerged as a functionally relevant component of adhesion-stress SGs. Prior work has shown that SGs can limit apoptosis through sequestration or regulation of apoptotic signaling factors[17–19]. In contrast to executioner caspases such as caspase-3 and caspase-7, CASP4 was selectively enriched in FAK inhibitor-induced SGs in our proteomic dataset. Loss of G3BP-dependent SG assembly increased CASP4 activation during FAK inhibitor-induced adhesion stress, and CASP4 knockdown partially rescued the survival defect of G3BP1/2-deficient MDA-MB-468 cells under anchorage-independent conditions. The mechanism by which SGs limit CASP4 activation remains to be defined. Our detection of full-length CASP4 in SG-associated fractions suggests that SGs may retain CASP4 before its activation, although further work will be needed to determine how this affects CASP4 processing and downstream signaling.

Our findings also separate two related functions of PERK during adhesion stress. PERK acts upstream of eIF2α phosphorylation and SG assembly, but it also contributes to the maintenance of AKT–mTOR survival signaling during prolonged anoikis in PTEN-deficient breast cancer cells. This dual function is consistent with the broader role of PERK in tumor adaptation, invasion, and metastatic progression[32–34, 50–52]. In our model, SG assembly limits CASP4 activation and apoptotic signaling, whereas PERK also helps sustain AKT–mTOR activity during anchorage-independent stress. Thus, adhesion-stress signaling coordinates translational stress adaptation with survival pathway maintenance.

G3BP-dependent SG assembly supported several cancer cell fitness traits, including proliferation, metabolic stress tolerance, anchorage-independent survival, and migration. These findings are consistent with previous work linking SG proteins and SG-associated pathways to tumor cell proliferation and stress adaptation[53–58]. Stress-response pathways that intersect with SG biology have been implicated in mitotic regulation[59], and G3BP1-containing ribonucleoprotein complexes have recently been linked to focal adhesion protein mobility and cell migration[60]. Nevertheless, these phenotypes should be interpreted with some caution. G3BP and other SG-associated proteins can act both within and outside SGs, and individual SG components did not contribute equally across the proliferation, survival, and migration assays in our study. Future work will be needed to define which effects reflect SG-dependent RNA or protein compartmentalization and which reflect non-granular functions of G3BP proteins or other SG-associated factors.

The enrichment of SG-positive cells in invasive regions of human breast cancer specimens provides supportive tissue-level evidence for adhesion-stress SGs in an invasive context. SGs were increased in pan-keratin-positive tumor cells within invasive regions and were most prominent in singly invading cancer cells. These observations are consistent with the idea that invading tumor cells encounter altered adhesion cues and activate stress-adaptive mechanisms during tissue invasion. Future studies using larger patient cohorts and spatially resolved molecular analyses will help determine whether adhesion-stress SGs associate with specific invasion patterns, tumor subtypes, therapeutic responses, or clinical outcomes.

Finally, our findings suggest that SG assembly may represent a targetable adaptive response during adhesion-directed therapy. Genetic loss of G3BP1/2 increased sensitivity to FAK inhibition, and pharmacologic inhibition of G3BP-dependent SG assembly enhanced the response to multiple FAK inhibitors in a cell context-dependent manner. We do not suggest that SG inhibition will be uniformly effective across all tumor contexts. The combination response varied among breast cancer cell lines, likely reflecting differences in baseline FAK dependence, PI3K–AKT–mTOR activity, SG assembly capacity, and other survival pathways. Defining molecular features that predict sensitivity to combined FAK and SG inhibition will be important for future work.

In summary, impaired adhesion engages a RhoA–ROCK–PERK–eIF2α stress pathway that couples adhesion loss to SG assembly. Adhesion-stress SGs form a distinct Rho GTPase-associated SG subtype that limits CASP4 activation and supports anchorage-independent survival. In parallel, PERK helps maintain AKT–mTOR survival signaling during anoikis. This framework positions SGs as adaptive survival assemblies during loss of adhesion and suggests that disrupting SG assembly may increase the vulnerability of adhesion-stress-adapted cancer cells to FAK-directed therapies.

## Materials and methods

### Cell culture and cell lines

U2OS, MEF, MDA-MB-468, SK-BR-3, MCF7, MDA-MB-361, MDA-MB-231, HeLa, MCF10A, and HAP1 cells were maintained at 37°C in a humidified incubator with 5% CO_2_. Unless otherwise indicated, cells were cultured in DMEM (Corning) supplemented with 10% fetal bovine serum (FBS), 100 U/ml penicillin, and 100 μg/ml streptomycin. Wild-type and knockout cell lines, including U2OS WT and G3BP1/2 KO cells, WT and ΔG3BP1 MEFs, MDA-MB-468 WT, G3BP1/2 KO, ΔGCN2, ΔPKR, ΔHRI, and ΔPERK cells, and HAP1 WT and ΔPERK cells, were generated and described in previous studies[61, 62].

### 7-Methyl GTP Sepharose chromatography

The eIF4F complex was captured using m^7^GTP–Sepharose beads as described previously[63]. In brief, m^7^GTP–Sepharose beads were washed twice with ice-cold 1X immunoprecipitation (IP) buffer (Cell Signaling, #9803) containing 20 mM Tris-HCl (pH 7.5), 150 mM NaCl, 1 mM Na_2_EDTA, 1 mM EGTA, 1% Triton X-100, 2.5 mM sodium pyrophosphate, 1 mM β-glycerophosphate, 1 mM Na_3_VO_4_, 1 μg/ml leupeptin, and 1 mM PMSF. U2OS cells were treated with the indicated inhibitors and harvested in IP buffer. Cell lysates were clarified by centrifugation, and equal amounts of lysate supernatant were incubated with pre-washed m^7^GTP–Sepharose beads at 4°C for 1 h with rotation. Unbound proteins were removed by washing the beads three times with IP buffer. Proteins bound to the beads were eluted using SDS sample buffer and analyzed by immunoblotting.

### Immunoblotting

Immunoblotting was performed as described previously[27, 64, 65]. Cells were washed twice with ice-cold PBS containing 1 mM sodium fluoride, 1 mM Na_3_VO_4_, and 1 mM PMSF to remove residual FBS and preserve phosphorylation during cell harvesting. Cells were lysed in Cell Lysis Buffer (Cell Signaling, #9803) containing 20 mM Tris-HCl (pH 7.5), 150 mM NaCl, 1 mM Na_2_EDTA, 1 mM EGTA, 1% Triton X-100, 2.5 mM sodium pyrophosphate, 1 mM β-glycerophosphate, 1 mM Na_3_VO_4_, 1 μg/ml leupeptin, and 1 mM PMSF, supplemented with either 1% SDS or RIPA buffer (Cell Signaling, #9806). Protein concentrations were determined, and equal amounts of protein were separated by SDS-PAGE and transferred to nitrocellulose membranes. Membranes were incubated with primary antibodies in PBS containing 5% normal horse serum overnight at 4°C, followed by washing with TBS-T. Membranes were then incubated with secondary antibodies in PBS containing 5% normal horse serum for 1 h at room temperature. For the p-MLC antibody, secondary antibody incubation was performed in TBS-T containing 5% skim milk.

### GFP-Trap pull-down assay and mass spectrometry

U2OS G3BP1/2 KO cells were transfected with plasmids encoding EGFP-empty, EGFP-G3BP1, or EGFP-G3BP1-ΔNTF2 using GenJet Plus DNA In Vitro Transfection Reagent (Signagen, #SL100499). Cells were harvested 48 h after transfection under the indicated treatment conditions. Cells were washed twice with ice-cold PBS and lysed in 1X IP buffer (Cell Signaling, #9803). ChromoTek GFP-Trap Magnetic Agarose beads (Proteintech, #gtma) were washed twice with ice-cold 1X IP buffer using a magnetic stand. Clarified cell lysates were incubated with the beads at 4°C for 1 h with rotation. After three washes with IP buffer, bound proteins were eluted using SDS sample buffer. G3BP1-associated proteins were analyzed by immunoblotting or by mass spectrometry at the Taplin Mass Spectrometry Facility, Harvard Medical School, Boston, MA.

### siRNA transfection

Cells were transfected with siRNAs targeting a negative control sequence (siNC; Ambion, Invitrogen, Cat# 4390843) or CASP4 using siCASP4-1 (ID: s2414) and siCASP4-2 (ID: s2413). Transfections were performed using PepMute siRNA Transfection Reagent (Signagen) according to the manufacturer’s instructions. Cells were seeded at approximately 50% confluence before transfection. Gene silencing was assessed 48–72 h after transfection.

### Pathway analysis

Gene ontology (GO), KEGG, and Reactome pathway enrichment analyses were performed using Metascape (https://metascape.org/) as described previously[65, 66]. For stress granule proteomic analyses, FAK14-selective, sodium arsenite-selective, and shared SG-associated protein sets were analyzed separately where indicated.

### Stress granule formation with FAK inhibitors

Stress granule formation following FAK inhibition was induced using FAK Inhibitor 14 (FAK14; Tocris, Cat# 3414; Sigma, Cat# SML0837). FAK14 was dissolved in DMSO as a 50 mM stock solution. To maintain inhibitor activity, stocks were aliquoted and repeated freeze–thaw cycles were avoided. Cells were treated with FAK inhibitor by replacing the culture medium with fresh medium containing the indicated concentration of inhibitor. DMSO-treated cells were used as vehicle controls.

### Migration assay

Migration assays were performed as described previously[64, 65]. Cells were suspended in 150 μl serum-free medium and seeded onto 8-μm pore Transwell inserts (Corning, #353097). The lower chamber contained 900 μl medium supplemented with either 10% or 0.5% FBS, as indicated. After 24 h, cells were fixed with 4% paraformaldehyde for 30 min and stained with hematoxylin (Sigma-Aldrich, #MHS16) for 2 h. Cells remaining on the upper surface of the membrane were removed using cotton swabs. Migrated cells on the lower surface were imaged by light microscopy and manually counted. Mean migrated cell numbers were calculated from randomly selected fields of equal area per insert. For relative migration assays, the number of migrated WT or control cells was set to 1, and values from experimental groups were normalized accordingly.

### Anoikis assay

Poly-HEMA-coated dishes were used as a model substrate for anoikis assays as described previously[1, 27, 64]. Briefly, a 10 mg/ml solution of poly-HEMA (Sigma-Aldrich, #P3932) prepared in 95% ethanol was added to cell culture dishes and dried in a tissue culture hood. Once completely dried, dishes were washed extensively with PBS at least twice before use. Cells were seeded onto poly-HEMA-coated dishes and incubated for the indicated time periods. Cell Counting Kit-8 (CCK-8; Abcam, #ab228554) reagent was added directly to the poly-HEMA-coated dishes containing suspended cells. Dishes containing medium without cells were included as negative controls for background subtraction. Relative cell number or viability signal was measured according to the manufacturer’s protocol, as described previously[27, 64]. Final CCK-8 values were normalized to values obtained from cells at the initial seeding time point or to the indicated control group.

### Cell proliferation and viability under metabolic stress

Cell proliferation and viability under metabolic stress were assessed using the CCK-8 assay (Abcam, #ab228554) as described previously[27, 64, 65]. Cells were seeded and allowed to attach firmly for 1 day before treatment. Attached cells were washed twice with Hank’s Balanced Salt Solution containing calcium and magnesium (HBSS with Ca^2+^/Mg^2+^) and then incubated in the indicated culture conditions, including 10% FBS medium, 0.5% FBS medium, or HBSS with Ca^2+^/Mg^2+^. CCK-8 reaction buffer was prepared in 5% FBS DMEM, and was used to measure CCK-8 values. The final CCK-8 values were normalized by the CCK-8 values obtained from early seeded cells as the starting point CCK-8 values.

### Calculation of relative cell number, viability, proliferation, and migration

For CCK-8 assays, cells were seeded in either 96-well or 24-well plate formats. Following treatment, CCK-8 solution was added at 5–10% of the total medium volume, depending on the assay format. For 96-well assays, CCK-8 reagent was added directly to the existing medium to avoid cell loss during medium replacement. This format was used for cell proliferation, cell viability, and anoikis resistance assays, including assays performed with or without poly-HEMA coating. Cells were incubated with CCK-8 reagent at 37°C for 1 h.

For 24-well assays used in proliferation and metabolic stress experiments, cells were seeded and allowed to adhere completely for 1 day. Following treatment, the existing culture medium was removed, and cells were incubated with fresh reaction medium containing 5–10% CCK-8 solution. After a 1 h incubation, the reacted medium was transferred to a 96-well plate for absorbance measurement. Absorbance was measured at 450 nm using a microplate reader. Wells containing medium without cells were included as blank controls, and blank values were subtracted from the absorbance values obtained from wells containing cells. Relative cell numbers were calculated by normalizing the data to the untreated vehicle group, WT cells, or the indicated control group, which was set to 1.

For Calcein AM live-cell viability assays, cells were seeded in 96-well black/clear-bottom plates. Following treatment, Calcein AM was added directly to the cells at a final concentration of 2 μM. Untreated cells were used as 100% viable controls, and wells containing medium without cells were used as blanks. Fluorescence values were background-subtracted using blank wells and used to calculate relative survival after FAK inhibitor treatment.

For Ki-67 staining assays, cells were seeded on coverslips coated with 10 μg/ml fibronectin for 1 h to allow complete cell adhesion. Medium was then replaced with 10% FBS medium. After 1 day, cells were fixed and stained with Ki-67 and Hoechst. Cells strongly positive for Ki-67 were counted and calculated as a percentage of the total cell number. Values were normalized to the indicated control group, which was set to 1.

For Transwell migration assays, migrated cells were counted from randomly selected fields of equal area. The number of migrated WT or control cells was set to 1, and relative migration values were calculated for knockout or treatment groups. Unless otherwise indicated, all quantification experiments were performed with at least three independent biological replicates.

### RhoA GTPase activation assay

RhoA activation was measured using a colorimetric RhoA G-LISA Activation Assay Kit (Cytoskeleton, Cat# BK124) according to the manufacturer’s protocol[27]. Cells were lysed, and protein concentrations were determined. Equal amounts of protein were added to a G-LISA plate pre-coated with Rho-GTP-binding protein. After incubation, wells were washed, and an antibody specific for RhoA was added. Following additional washing, secondary antibody conjugated to a detection reagent was applied. Absorbance was measured using a microplate reader and used to determine relative RhoA activation levels.

### Caspase-4 activation assay

Caspase-4 activity was measured in WT and G3BP1/2 KO cells using a Caspase-4 Colorimetric Assay Kit (Cat# MBS9718956) according to the manufacturer’s protocol. Cells were treated with or without FAK14 (25 μM) for 24 h. After treatment, cells were harvested and lysed. Protein concentrations were determined using Precision Red Advanced Protein Assay Reagent (Cytoskeleton, Cat# ADV02). Equal amounts of protein from each lysate were used for the Caspase-4 activity assay. Samples were incubated with the Ac-LEVD-pNA substrate, and absorbance was measured using a microplate reader. Caspase-4 activity was calculated from a pNA standard curve and normalized to the indicated control group.

### Human breast tumor samples

A tissue microarray containing human breast and breast cancer tissues was obtained from TissueArray (TissueArray, #BR087e). Tissue sections were used for immunofluorescence analysis of stress granule markers and epithelial tumor-cell markers as described below.

### Ribopuromycylation

Ribopuromycylation was performed as described previously[61]. Briefly, puromycin was added directly to the culture medium at a final concentration of 9 μM 5 min before cell harvest. Cells were incubated with puromycin for 5 min at 37°C and then harvested for standard immunoblotting. Puromycin incorporation was detected using an anti-puromycin antibody (Millipore, #MABE343, clone 12D10, IgG2a).

### Immunofluorescence analysis of cells and tissues

Immunofluorescence analysis was performed as described previously[27, 61, 64, 65]. For immunofluorescence analysis of cultured cells, cells grown on slide glass or coverslips were fixed with 4% paraformaldehyde in PBS for 30 min and permeabilized with 1% Triton X-100 (Sigma-Aldrich, #T8787) in PBS for 20 min. Cells were then washed with PBS containing 0.1% Triton X-100. Samples were blocked with 1% albumin from chicken egg white (Sigma-Aldrich, #A5503) in IF washing buffer for 1 h. Cells were incubated with primary antibodies in IF washing buffer for 1 h, followed by three washes for 10 min each. Cells were then incubated with secondary antibodies together with Rhodamine Phalloidin Reagent or Phalloidin FITC Reagent (Abcam, #ab235138 or #ab235137) in IF washing buffer for 1 h. Samples were washed three times for 10 min each, and Hoechst 33258 (Sigma-Aldrich, #H21486) was added during the second wash.

For immunofluorescence analysis of tissues, formalin-fixed, paraffin-embedded tissue sections were deparaffinized and rehydrated. Tissue slides were placed on a slide warmer at 62°C for 20 min. Slides were then immersed in 100% xylene three times for 3 min each, followed by immersion in 100% ethanol three times for 3 min each. Slides were serially rehydrated in 95% ethanol for 3 min, 70% ethanol for 3 min, and 50% ethanol for 3 min, and then rinsed in water for 5 min. For antigen retrieval, slides were incubated in Tris-EDTA buffer containing 1.27 ml of 1 M EDTA, 5 ml of 2 M Tris-HCl, and 0.5 ml Tween 20 per liter of H_2_O, adjusted to pH 7.5, at 90–95°C for 20 min. Slides were rinsed in cold tap water for 10 min. Tissue sections were blocked with 1% albumin from chicken egg white in IF washing buffer for 1 h and then incubated with primary antibodies in IF washing buffer for 1 h. Slides were washed three times for 10 min each, incubated with secondary antibodies in IF washing buffer for 1 h, and washed again three times for 10 min each. Hoechst 33258 was added during the second wash.

### Statistical analysis

Statistical analyses were performed using Prism 10 (GraphPad). Unless otherwise indicated, experiments were performed with at least three independent biological replicates. Sample sizes (n) represent biologically independent replicates, such as independent cell cultures, independent samples, or independently analyzed fields, as indicated in the figure legends. For graphs, individual data points represent independent samples. Statistical significance was assessed using Student’s t-test or ANOVA, as appropriate for the experimental design. P values < 0.05 were considered statistically significant. Statistical significance is indicated as *P < 0.05, **P < 0.01, and ***P < 0.001. Representative micrographs and immunoblots are shown from experiments that were independently repeated with similar results.

## Supplementary Material

1

Supplementary Files

This is a list of supplementary files associated with this preprint. Click to download.

- SYMetascaperesultFAK14only12706082026.xlsx
- SYMetascaperesultSAonly18906082026.xlsx
- SYMetascaperesultSAFAK14shared12706082026.xlsx
- SYPathwayanalysis.xlsx
- SYSupplementalVideoA06082026.mp4
- SYSupplementalVideoB06082026.mp4
- SYSourcedata06082026.xlsx
- SYSummaryofProteomicanalysis.xlsx

## Figures and Tables

**Fig. 1.**
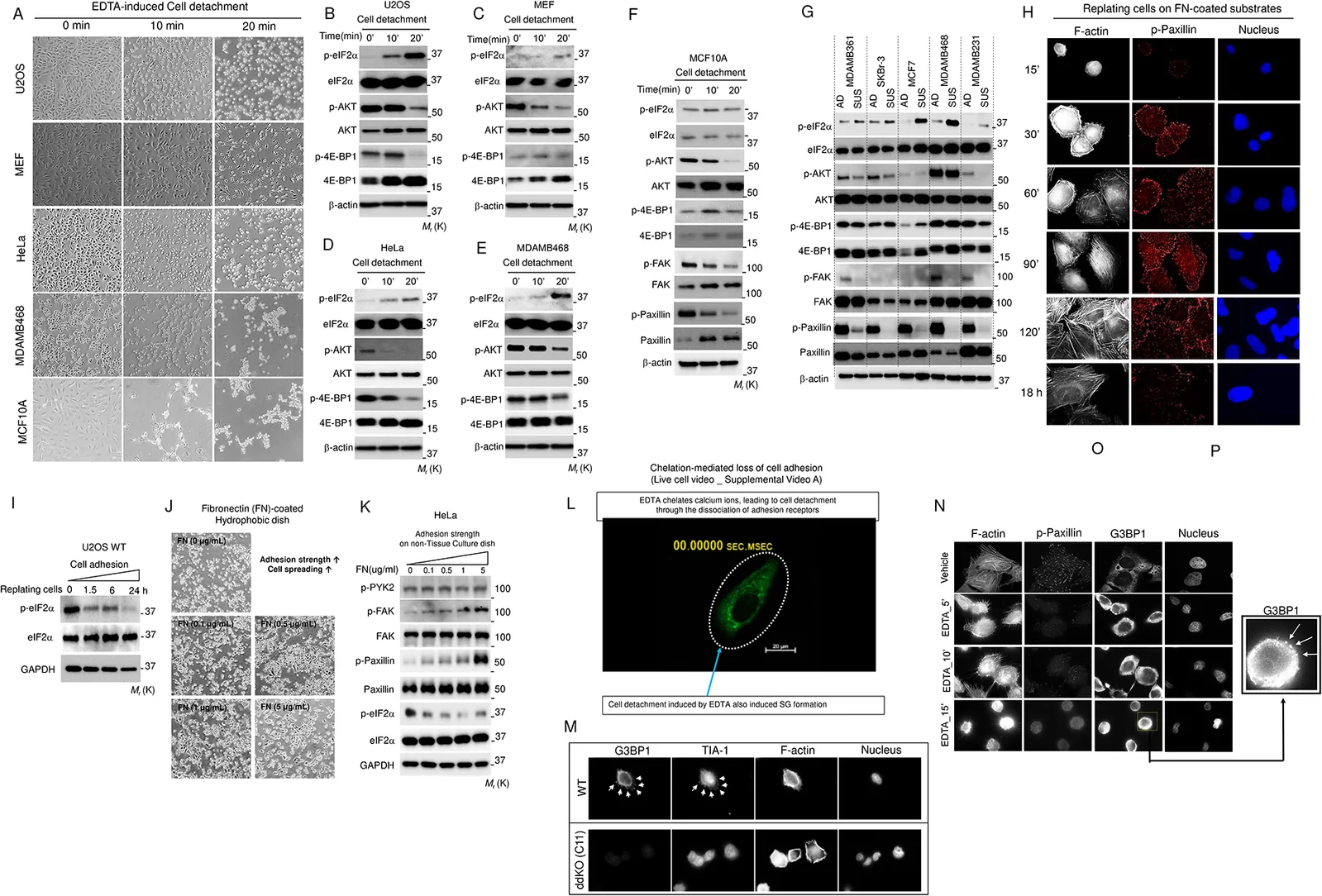
Loss of adhesion induces eIF2α phosphorylation and induces SG assembly. (A) Representative phase-contrast images showing EDTA-induced detachment of U2OS, MEF, HeLa, MDA-MB-468, and MCF10A cells over time. EDTA was added directly to the culture medium at a final concentration of 2 mM to induce chelation-mediated loss of adhesion. (B–F) Immunoblot analysis of U2OS, MEF, HeLa, MDA-MB-468, and MCF10A cells during 2 mM EDTA-induced detachment. Cells were harvested at the indicated time points after EDTA addition and analyzed for phosphorylation of eIF2α, AKT, 4E-BP1, FAK, and paxillin, together with the corresponding total proteins and loading controls. (G) Immunoblot analysis of adherent and suspension breast cancer cells after 2 mM EDTA-induced detachment. MDA-MB-361, SK-BR-3, MCF7, MDA-MB-468, and MDA-MB-231 cells were analyzed under adherent and suspension conditions for phosphorylation of eIF2α, AKT, 4E-BP1, FAK, and paxillin. (H) Representative immunofluorescence images showing U2OS cell spreading during replating on fibronectin-coated substrates. Cells were fixed at the indicated time points after replating and stained for F-actin, phospho-paxillin, and nuclei. (I) Immunoblot analysis of eIF2α phosphorylation during U2OS cell replating. Cells were collected at the indicated time points after replating on fibronectin-coated hydrophobic dishes. (J) Representative phase-contrast images of HeLa cells seeded on fibronectin-coated hydrophobic dishes to modulate adhesion strength. Increasing fibronectin concentrations increased cell spreading. (K) Immunoblot analysis of adhesion signaling and eIF2α phosphorylation in HeLa cells seeded on fibronectin-coated hydrophobic dishes. Cells were plated on dishes coated with the indicated concentrations of fibronectin and analyzed after adhesion. (L) Representative frame from live-cell imaging of EDTA-induced detachment in U2OS G3BP1/2 KO cells expressing EGFP-G3BP1. EDTA-induced loss of adhesion was accompanied by rapid EGFP-G3BP1 granule formation. (M) Representative immunofluorescence images showing G3BP1- and TIA-1-positive SG formation during early replating on fibronectin-coated substrates. WT and G3BP1/2-deficient cells were replated onto fibronectin-coated coverslips and fixed during the early adhesion phase. F-actin staining was used to visualize cell shape and the extent of cell spreading during replating. G3BP1/2-deficient cells were included as an SG-deficient control.. (N) Representative immunofluorescence images showing focal adhesion staining and SG assembly during EDTA-induced detachment. Cells were fixed after vehicle or EDTA treatment for the indicated times and stained for F-actin, phospho-paxillin, G3BP1, and nuclei. White arrows indicate G3BP1-positive SGs. Numerous small G3BP1-positive puncta were observed near the cell periphery as cells lost adhesion and phospho-paxillin staining decreased. Representative images and immunoblots are shown from experiments independently repeated at least three times with similar results.

**Fig. 2.**
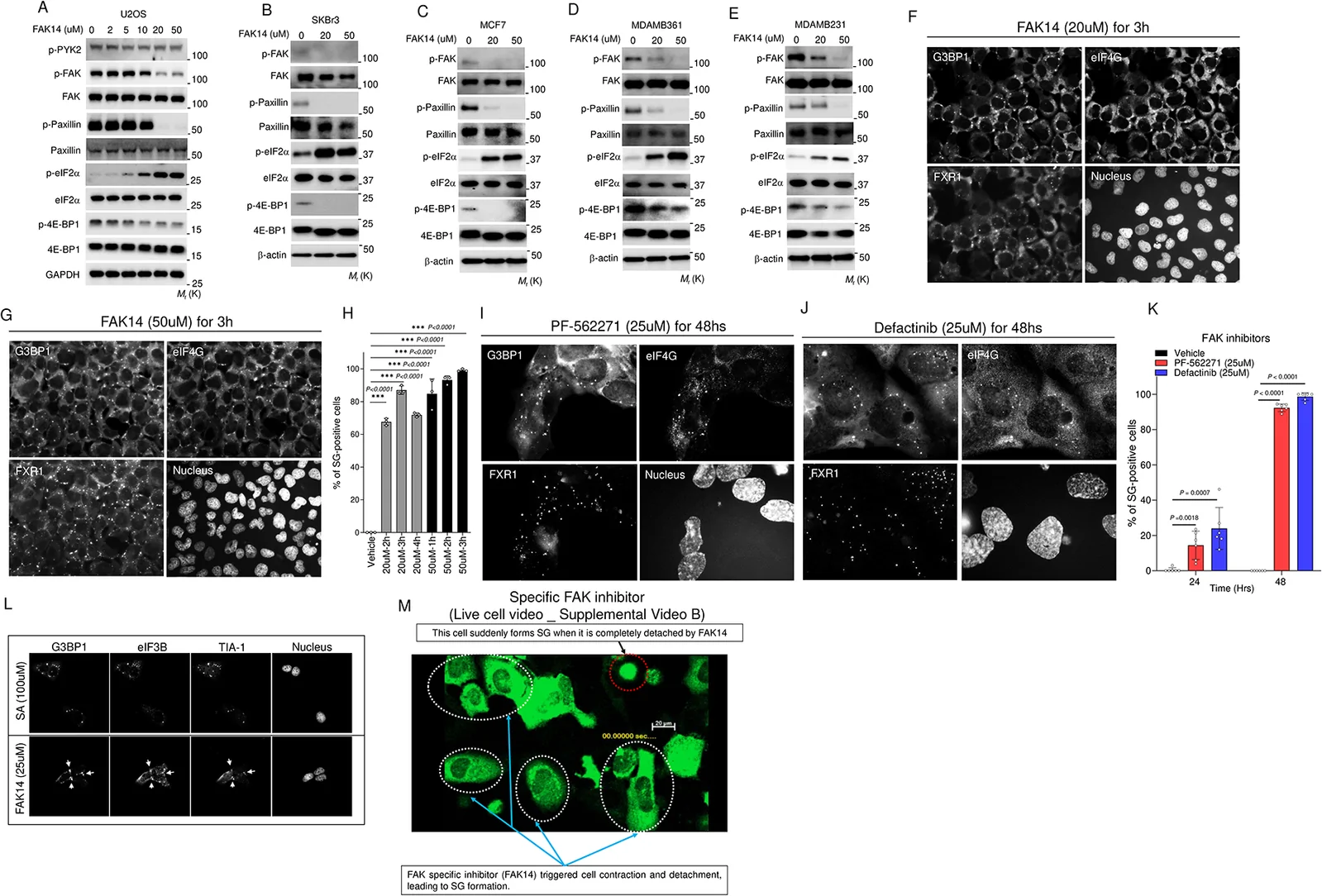
FAK inhibition induces canonical SG assembly during impaired adhesion signaling. (A) Immunoblot analysis of U2OS cells treated with increasing concentrations of FAK14 for 1 h. Phosphorylation of FAK and paxillin was used to monitor focal adhesion signaling, and phosphorylation of eIF2α and 4E-BP1 was examined as markers of translational stress signaling. PYK2 phosphorylation was included to assess whether acute FAK inhibition induced compensatory PYK2 activation. (B–E) Immunoblot analysis of SK-BR-3, MCF7, MDA-MB-361, and MDA-MB-231 cells treated with the indicated concentrations of FAK14 for 1 h. Focal adhesion signaling and translational stress-response markers were analyzed as in (A). (F,G) Immunofluorescence images of U2OS cells treated with FAK14 for 3 h and stained for G3BP1, eIF4G, FXR1, and nuclei. (H) Quantification of SG-positive U2OS cells after FAK14 treatment at the indicated concentrations and time points. Cells containing G3BP1-positive cytoplasmic granules were scored as SG-positive. (I,J) Immunofluorescence images of U2OS cells treated with additional FAK inhibitors. Cells were treated with PF-562271 (25 μM) or defactinib (25 μM) for 48 h and stained for G3BP1, eIF4G, FXR1, and nuclei. (K) Quantification of SG-positive cells after PF-562271 or defactinib treatment for the indicated times. (L) Immunofluorescence images comparing sodium arsenite-induced SGs with FAK14-induced SGs. Cells were stained for G3BP1, eIF3B, TIA-1, and nuclei to evaluate recruitment of canonical SG markers. (M) Representative frame from live-cell imaging of U2OS G3BP1/2 KO cells expressing EGFP-G3BP1 during FAK14 treatment. FAK14 induced cell contraction and detachment, and EGFP-G3BP1 granules formed as cells transitioned toward a detached morphology. Data are shown as mean ± SD. Statistical analysis was performed as described in Methods. Representative images and immunoblots are from experiments independently repeated at least three times with similar results.

**Fig. 3.**
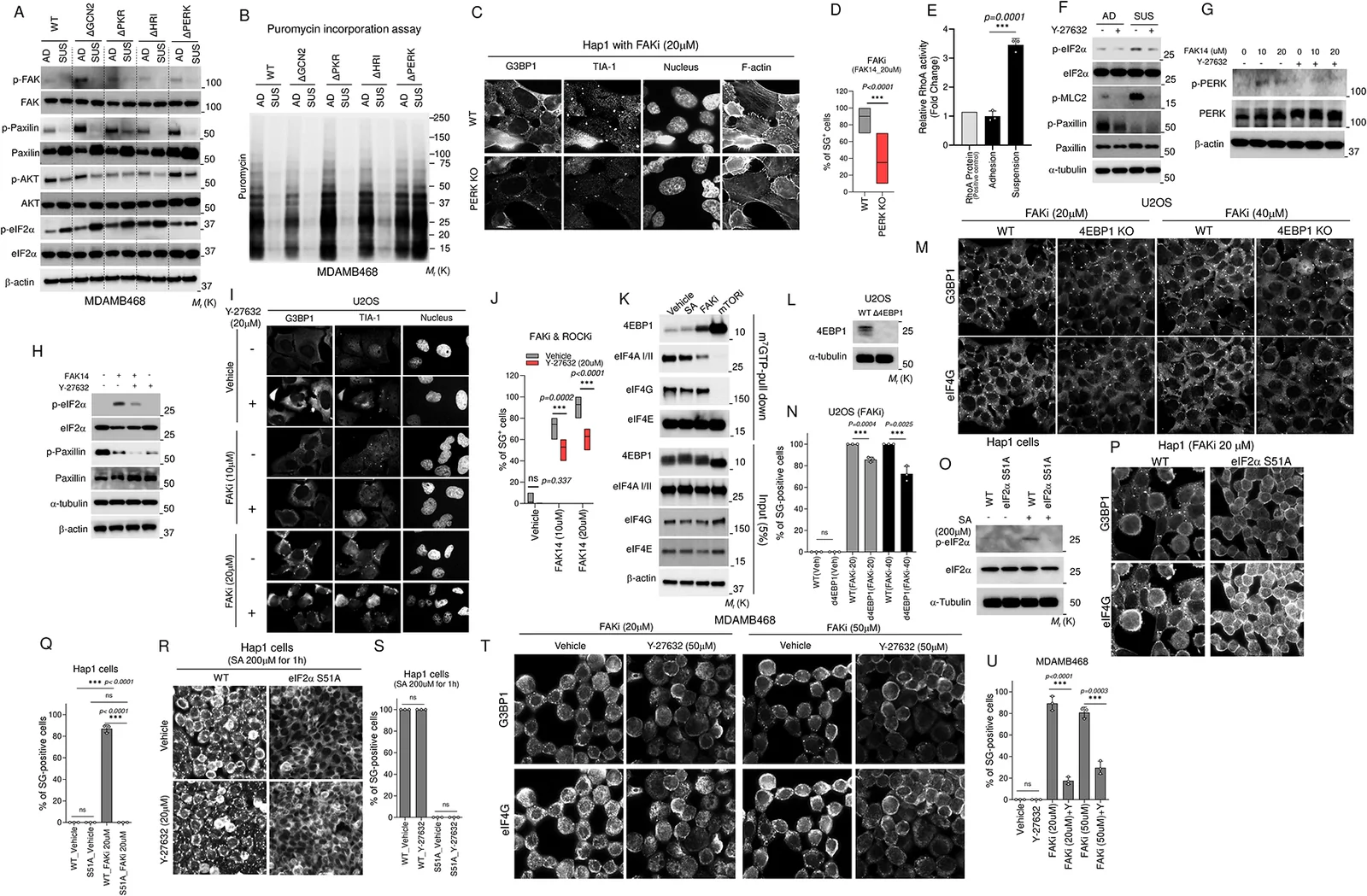
Adhesion stress activates a ROCK–PERK–eIF2α pathway that promotes SG assembly. (A) Immunoblot analysis of MDA-MB-468 WT, ΔGCN2, ΔPKR, ΔHRI, and ΔPERK cells under adherent and EDTA-induced suspension conditions. Cells were analyzed for eIF2α phosphorylation, focal adhesion signaling, and AKT signaling. (B) Puromycin incorporation assay in MDA-MB-468 WT and eIF2α kinase-deficient cells under adherent and suspension conditions. Puromycin incorporation was used to monitor global protein synthesis. (C,D) Immunofluorescence images and quantification of SG-positive cells in HAP1 WT and ΔPERK cells treated with FAK14. Cells were stained for G3BP1, TIA-1, F-actin, and nuclei. (E) RhoA activation assay in adherent and suspension cells. RhoA activity was measured using a colorimetric G-LISA assay after EDTA-induced detachment. Recombinant RhoA protein was included as a positive control. (F) Immunoblot analysis of HeLa cells treated with EDTA in the presence or absence of the ROCK inhibitor Y-27632. MLC2 phosphorylation was used as a readout of ROCK activity, and eIF2α phosphorylation was monitored during detachment. (G) Immunoblot analysis of U2OS cells treated with FAK14 in the presence or absence of Y-27632. PERK phosphorylation was analyzed to test whether ROCK activity lies upstream of PERK activation during FAK inhibitor-induced adhesion stress. (H) Immunoblot analysis of HeLa cells treated with FAK14 in the presence or absence of Y-27632. Cells were analyzed for focal adhesion signaling and eIF2α phosphorylation. (I,J) Immunofluorescence images and quantification of SG formation in U2OS cells treated with FAK14 in the presence or absence of Y-27632. Cells were stained for G3BP1, TIA-1, and nuclei. (K) m^7^GTP–Sepharose pull-down analysis of the cap-binding complex in U2OS cells treated with FAK14. Cap-associated 4E-BP1, eIF4A, eIF4G, and eIF4E were analyzed to examine eIF4F complex assembly. (L) Immunoblot analysis confirming loss of 4E-BP1 in U2OS 4E-BP1 KO cells. (M,N) Immunofluorescence images and quantification of FAK14-induced SG formation in U2OS WT and 4E-BP1 KO cells. Cells were stained for G3BP1 and eIF4G. (O) Immunoblot analysis of eIF2α phosphorylation in HAP1 WT and eIF2α S51A cells treated with sodium arsenite. (P,Q) Immunofluorescence images and quantification of FAK14-induced SG formation in HAP1 WT and eIF2α S51A cells. Cells were stained for G3BP1 and eIF4G. (R–U) Immunofluorescence images and quantification showing the effect of Y-27632 on sodium arsenite-induced SG formation in HAP1 cells. Cells were stained for G3BP1 and eIF4G to test whether ROCK inhibition broadly suppresses canonical oxidative stress-induced SG assembly. Data are shown as mean ± SD. Statistical analysis was performed as described in Methods. Representative images and immunoblots are from experiments independently repeated at least three times with similar results.

**Fig. 4.**
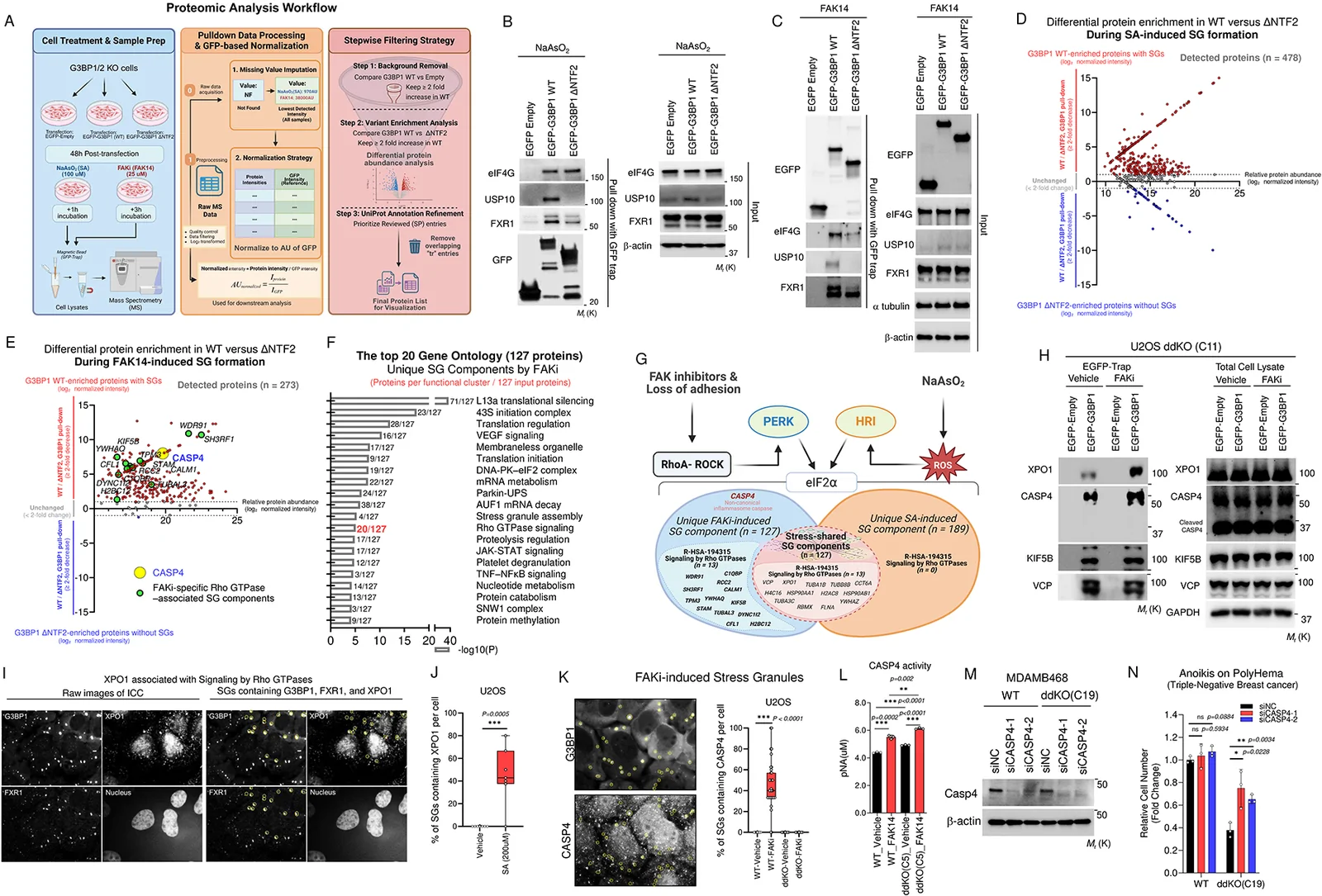
FAK inhibition induces a Rho GTPase-associated SG proteome enriched for CASP4. (A) Workflow for identifying SG-associated proteins after sodium arsenite or FAK14 treatment. U2OS G3BP1/2 KO cells were reconstituted with EGFP-empty, EGFP-G3BP1 WT, or EGFP-G3BP1-ΔNTF2. G3BP1-associated proteins were isolated by GFP-Trap pull-down and analyzed by mass spectrometry. Proteins preferentially recovered with EGFP-G3BP1 WT over EGFP-G3BP1-ΔNTF2 were considered SG assembly-dependent components. (B,C) Immunoblot analysis of GFP-Trap pull-down samples used for proteomic analysis after sodium arsenite or FAK14 treatment. eIF4G, USP10, and FXR1 were analyzed as representative SG-associated proteins in pull-down and input fractions. (D) Scatter plot showing G3BP1-associated proteins recovered with EGFP-G3BP1 WT or EGFP-G3BP1-ΔNTF2 during sodium arsenite-induced SG formation. Proteins enriched in the EGFP-G3BP1 WT pull-down were used to define sodium arsenite-induced SG assembly-dependent components. (E) Scatter plot showing G3BP1-associated proteins recovered with EGFP-G3BP1 WT or EGFP-G3BP1-ΔNTF2 during FAK14-induced SG formation. CASP4 and proteins linked to Rho GTPase-related pathways are highlighted. (F) Pathway analysis of FAK14-selective SG components. Enriched functional categories included Rho GTPase signaling, SG assembly, translation initiation, mRNA metabolism, and related pathways. (G) Classification of SG proteomic groups identified after sodium arsenite or FAK14 treatment. Shared, sodium arsenite-selective, and FAK14-selective SG-associated protein groups are indicated. (H) Immunoblot validation of selected FAK14-associated SG proteins recovered with EGFP-G3BP1. XPO1, KIF5B, CASP4, and VCP were analyzed in GFP-Trap pull-down and total cell lysate fractions. (I,J) Immunofluorescence images and quantification of XPO1 localization to SGs. Cells were treated with sodium arsenite and stained for G3BP1, FXR1, XPO1, and nuclei. (K) Immunofluorescence images and quantification of CASP4 localization to FAK14-induced SGs. Cells were stained for G3BP1, CASP4, and nuclei, and the percentage of CASP4-positive SGs was quantified. (L) Caspase-4 activity assay in U2OS WT and G3BP1/2 KO cells treated with vehicle or FAK14. Caspase-4 activity was measured using a colorimetric Ac-LEVD-pNA substrate assay. (M) Immunoblot analysis of CASP4 knockdown in MDA-MB-468 WT and G3BP1/2 KO cells transfected with control siRNA or two independent CASP4 siRNAs. (N) Anoikis assay in MDA-MB-468 WT and G3BP1/2 KO cells after CASP4 knockdown. Cells were cultured on poly-HEMA-coated dishes, and relative cell number was measured by CCK-8 assay. Data are shown as mean ± SD. Statistical analysis was performed as described in Methods. Representative images and immunoblots are from experiments independently repeated at least three times with similar results.

**Fig. 5.**
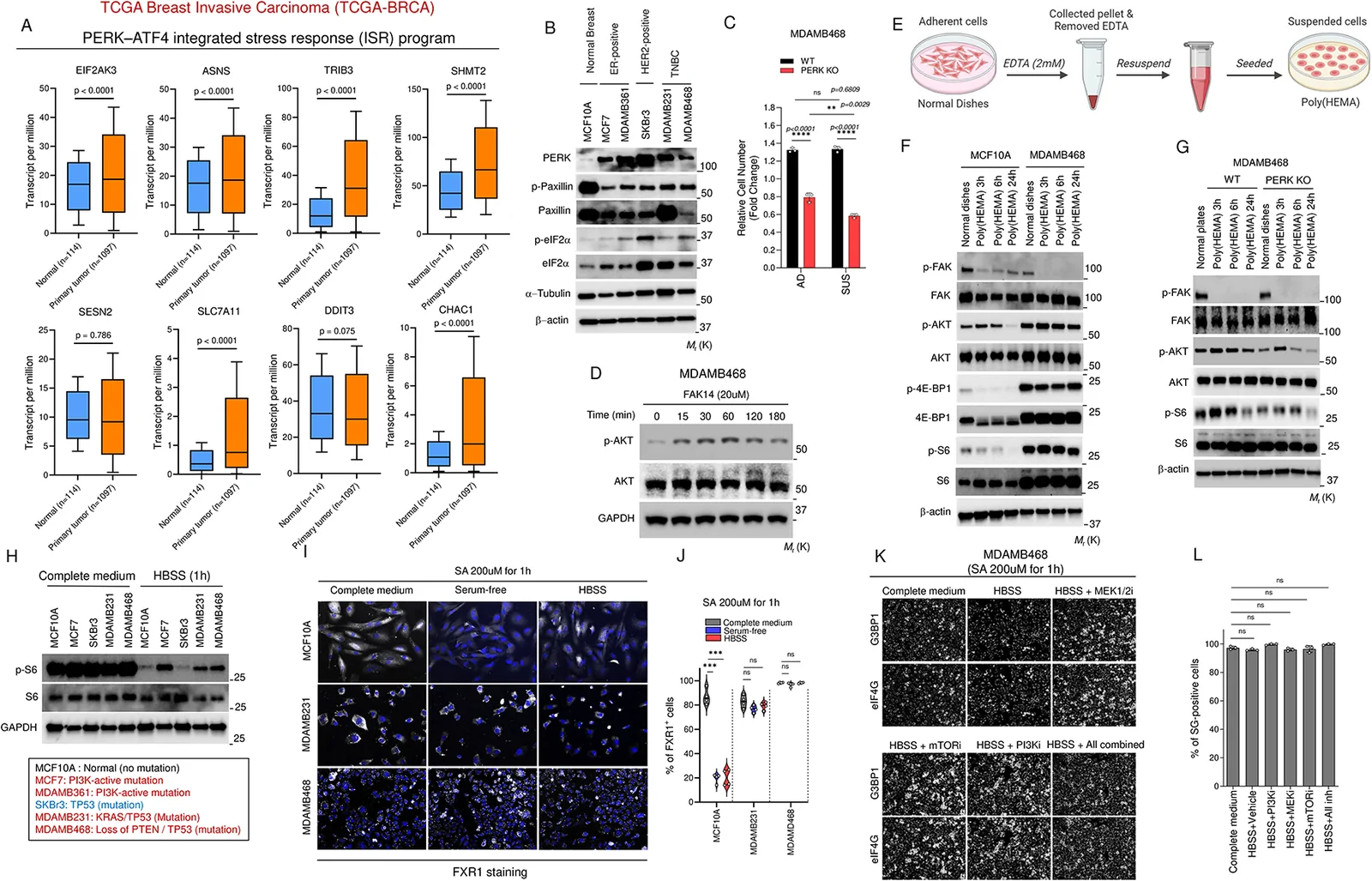
PERK supports AKT–mTOR signaling during anoikis. (A) Analysis of EIF2AK3 and PERK–ATF4 integrated stress response target genes in TCGA Breast Invasive Carcinoma samples. Transcript levels of EIF2AK3, ASNS, TRIB3, SHMT2, SESN2, SLC7A11, DDIT3, and CHAC1 were compared between normal breast tissue and primary breast tumors. (B) Immunoblot analysis of PERK, eIF2α phosphorylation, and adhesion-related signaling proteins in MCF10A cells and a panel of breast cancer cell lines. Cell-line subtype are indicated. (C) Relative cell number analysis of MDA-MB-468 WT and PERK KO cells under adherent and suspension conditions. Cells were cultured under the indicated conditions, and relative cell number was measured by CCK-8 assay. (D) Immunoblot analysis of AKT phosphorylation in MDA-MB-468 cells treated with FAK14 for the indicated times. (E) Schematic of the poly-HEMA suspension culture assay used to model anoikis. Cells were detached and replated on poly-HEMA-coated dishes to maintain suspension culture. (F) Immunoblot analysis of MCF10A and MDA-MB-468 cells cultured on normal dishes or poly-HEMA-coated dishes for the indicated times. FAK, AKT, 4E-BP1, and S6 phosphorylation were analyzed during suspension culture. (G) Immunoblot analysis of MDA-MB-468 WT and PERK KO cells cultured on normal dishes or poly-HEMA-coated dishes for the indicated times. AKT and S6 phosphorylation were analyzed to test whether PERK is required to maintain AKT–mTOR signaling during anoikis. (H) Immunoblot analysis of S6 phosphorylation in MCF10A and breast cancer cell lines under complete medium or HBSS conditions. Relevant mutation status is indicated. HBSS containing Ca^2+^ and Mg^2+^ was used as a nutrient-deprivation condition. The blot exposure was selected to visualize residual p-S6 signal under HBSS conditions; complete medium samples are shown as reference for basal p-S6 activity. (I,J) Immunofluorescence images and quantification of sodium arsenite-induced SG formation in MCF10A, MDA-MB-231, and MDA-MB-468 cells under complete medium or HBSS conditions. Cells were stained for FXR1 and nuclei. (K,L) Immunofluorescence images and quantification of SG formation in MDA-MB-468 cells under HBSS conditions after inhibition of MEK1/2, PI3K, mTOR, or all three pathways. Sodium arsenite was used to induce SG formation, and cells were stained for G3BP1 and eIF4G. Data are shown as mean ± SD. Statistical analysis was performed as described in Methods. Representative images and immunoblots are from experiments independently repeated at least three times with similar results.

**Fig. 6.**
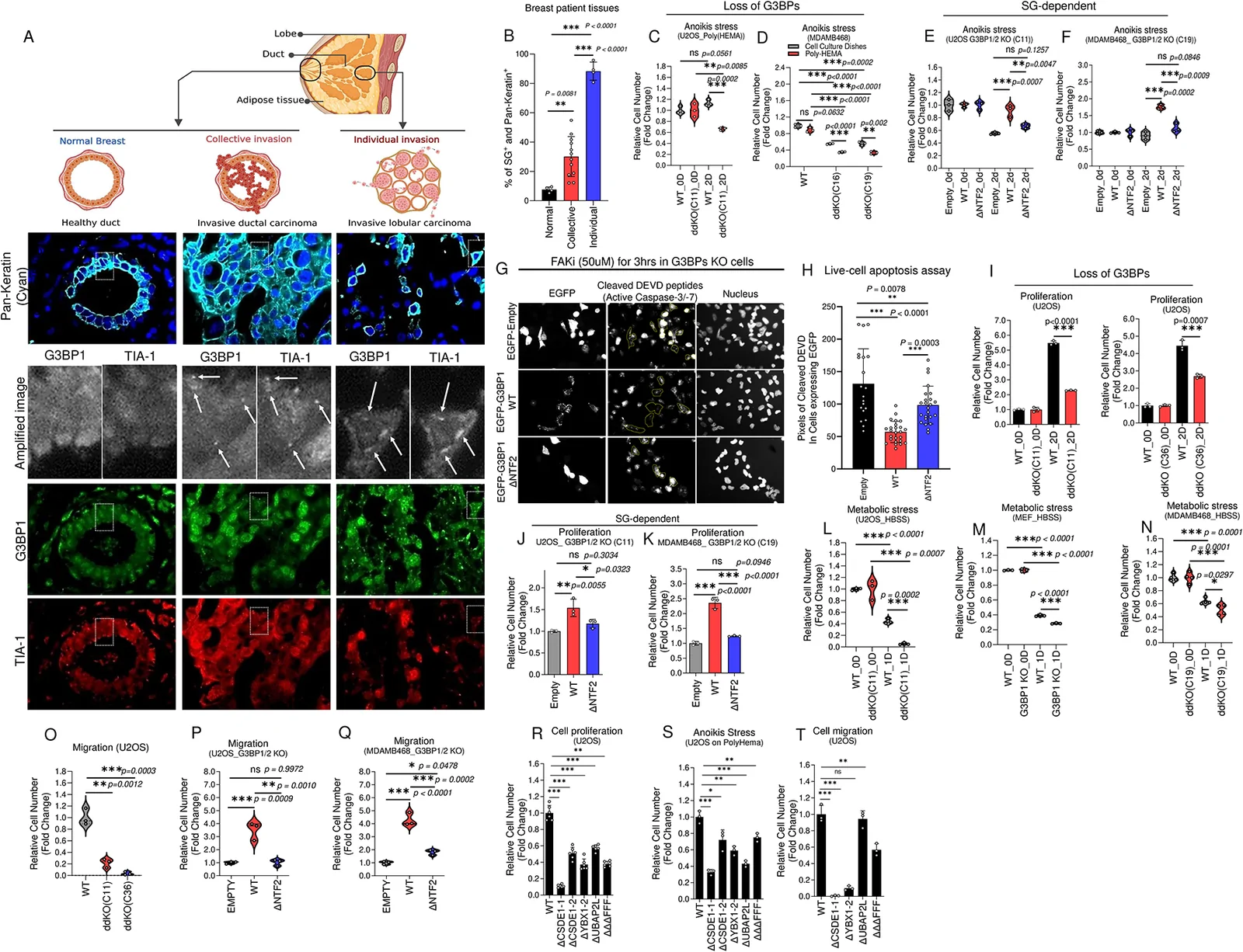
G3BP-dependent SG assembly supports anoikis resistance, proliferation, and migration. (A) Immunofluorescence images of human breast tissue and breast cancer specimens stained for pan-keratin, G3BP1, TIA-1, and nuclei. Pan-keratin was used to identify epithelial tumor cells, and G3BP1 and TIA-1 were used as SG markers. Enlarged images show G3BP1- and TIA-1-positive cytoplasmic puncta in pan-keratin-positive cells within invasive tumor regions. (B) Quantification of SG-positive, pan-keratin-positive cells in normal breast tissue, collective invasion regions, and individual invasion regions. Each point represents an independently analyzed tissue sample or tumor region. (C,D) Anoikis assays in U2OS WT and G3BP1/2 KO cells and in MDA-MB-468 WT and G3BP1/2 KO cells. Cells were cultured on poly-HEMA-coated dishes, and relative cell number was measured by CCK-8 assay at the indicated time points. (E,F) Anoikis rescue assays in U2OS and MDA-MB-468 G3BP1/2 KO cells expressing EGFP-empty, EGFP-G3BP1 WT, or EGFP-G3BP1-ΔNTF2. Cells were cultured on poly-HEMA-coated dishes, and relative cell number was measured at day 0 and day 2. (G,H) Live-cell apoptosis assay in U2OS G3BP1/2 KO cells expressing EGFP-empty, EGFP-G3BP1 WT, or EGFP-G3BP1-ΔNTF2 after FAK14 treatment. Active caspase-3/7 was detected using a cleaved DEVD fluorescent reporter, and signal intensity was quantified in EGFP-positive cells. (I) Proliferation assays in U2OS WT and G3BP1/2 KO cells and in MDA-MB-468 WT and G3BP1/2 KO cells. Relative cell number was measured by CCK-8 assay at the indicated time points. (J,K) Proliferation rescue assays in U2OS and MDA-MB-468 G3BP1/2 KO cells expressing EGFP-empty, EGFP-G3BP1 WT, or EGFP-G3BP1-ΔNTF2. (L–N) Metabolic stress assays in U2OS WT and G3BP1/2 KO cells, WT and ΔG3BP1 MEFs, and MDA-MB-468 WT and G3BP1/2 KO cells. Cells were incubated in HBSS containing Ca^2+^ and Mg^2+^, and relative cell number was measured by CCK-8 assay. (O) Transwell migration assay comparing U2OS WT and G3BP1/2 KO cells. Relative migration was calculated by normalizing migrated WT cells to 1. (P,Q) Migration rescue assays in U2OS and MDA-MB-468 G3BP1/2 KO cells expressing EGFP-empty, EGFP-G3BP1 WT, or EGFP-G3BP1-ΔNTF2. (R–T) Proliferation, anoikis, and migration assays in U2OS cells lacking additional SG-associated factors, including CSDE1, YB-1, UBAP2L, or FXR-family proteins. Relative cell number or migration was normalized to WT control cells. Data are shown as mean ± SD. Statistical analysis was performed as described in Methods. Representative images are from experiments independently repeated at least three times with similar results.

**Fig. 7.**
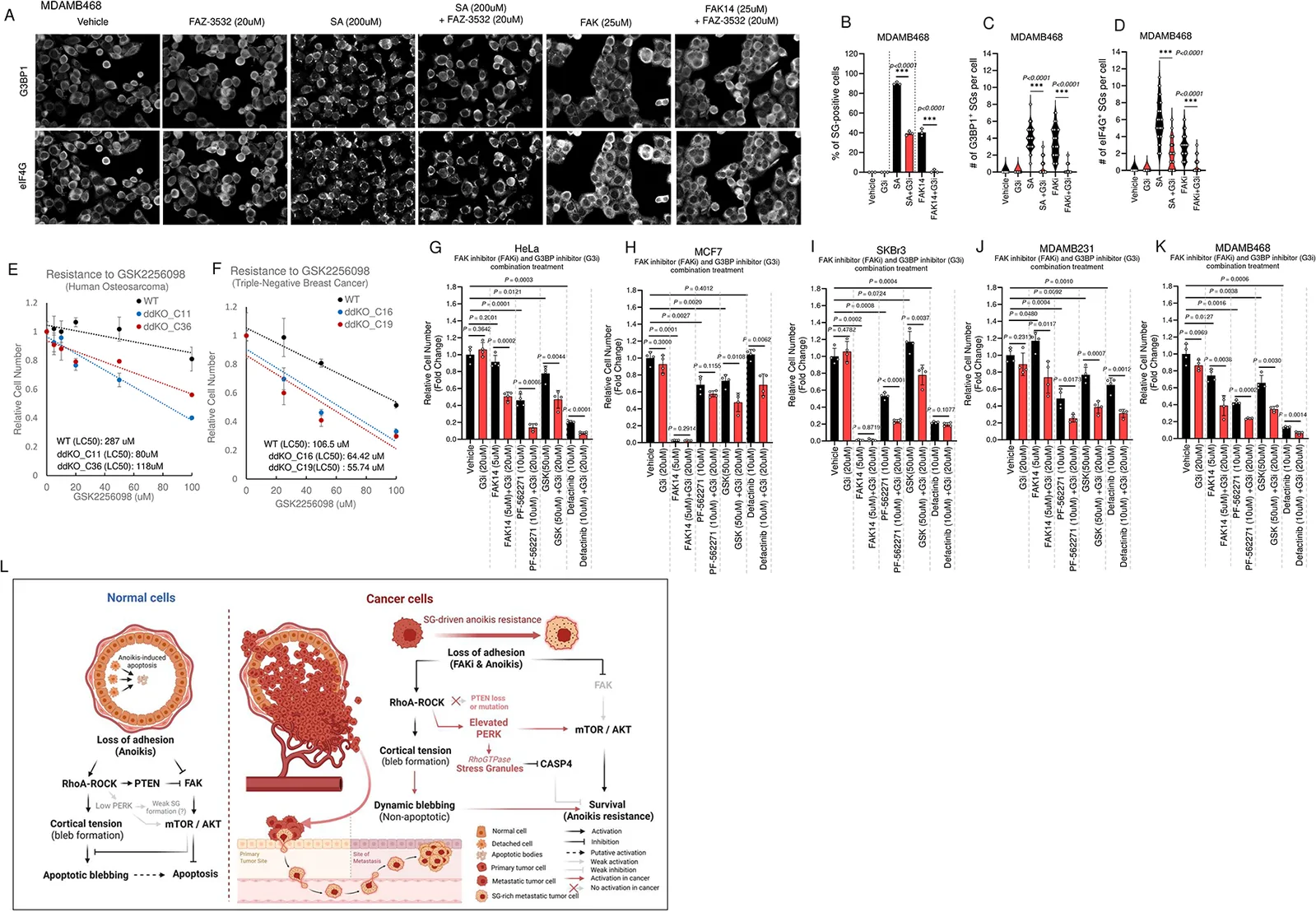
Inhibition of G3BP-dependent SG assembly sensitizes cells to FAK inhibition. (A) Immunofluorescence images showing the effect of FAZ-3532 on SG formation in MDA-MB-468 cells. Cells were treated with vehicle, FAZ-3532, sodium arsenite, sodium arsenite plus FAZ-3532, FAK14, or FAK14 plus FAZ-3532 and stained for G3BP1 and eIF4G. (B–D) Quantification of SG formation in MDA-MB-468 cells treated as in (A). SG-positive cells, G3BP1-positive SGs per cell, and eIF4G-positive SGs per cell were quantified. (E) Dose-response analysis of U2OS WT and G3BP1/2 KO cells treated with the FAK inhibitor GSK2256098. Relative cell number was measured after treatment, and LC50 values are indicated. (F) Dose-response analysis of MDA-MB-468 WT and G3BP1/2 KO cells treated with GSK2256098. Relative cell number was measured after treatment, and LC50 values are indicated. (G–K) Cell viability assays in HeLa, MCF7, SK-BR-3, MDA-MB-231, and MDA-MB-468 cells treated with FAK inhibitors alone or together with FAZ-3532. Cells were treated with FAK14, PF-562271, GSK2256098, or defactinib at the indicated concentrations, with or without FAZ-3532. Relative cell number was measured by CCK-8 assay and normalized to vehicletreated controls. (L) Working model. Loss of adhesion activates RhoA–ROCK–PERK–eIF2α signaling and promotes SG assembly. PERK also supports AKT–mTOR signaling during anoikis. Adhesion-stress SGs limit apoptotic signaling and CASP4 activation, thereby supporting anchorage-independent survival. Inhibition of G3BP-dependent SG assembly weakens this survival program and sensitizes cells to FAK inhibition. Data are shown as mean ± SD. Statistical analysis was performed as described in Methods. Representative images are from experiments independently repeated at least three times with similar results.

## Data Availability

The mass spectrometry proteomics data have been deposited to the ProteomeXchange Consortium via the PRIDE partner repository under the dataset identifier PXD058141. Other data from this study are available from the corresponding author or first author upon reasonable request.

## HIGHLIGHTS

- Adhesion stress triggers SG assembly to limit anoikis.
- A RhoA–ROCK–PERK–eIF2α pathway links impaired adhesion signaling to SG formation.
- Adhesion-stress SGs form a distinct Rho GTPase-associated SG subtype.
- SG assembly limits CASP4 activation, and PERK sustains AKT–mTOR survival signaling.
- Disrupting SG assembly increases anoikis sensitivity and enhances the response to FAK inhibition.
