# Supplementary material for: Rho GTPase–driven stress granules enable anchorage-independent survival under adhesion stress
Source: Res Sq. 2026 Oct 1:rs.3.rs-10284597. Preprint. [Version 1] doi: 10.21203/rs.3.rs-10284597/v1 (PMC13648863; doi:10.21203/rs.3.rs-10284597/v1)

Supplementary figure legends

Supplementary Fig. S1. Additional images of FAK inhibitor-induced SG assembly.

(A) Additional immunofluorescence images of U2OS cells treated with FAK14 at the indicated concentrations and times. Cells were stained for G3BP1, eIF4G, FXR1, and nuclei. These images correspond to the quantification shown in Fig. 2H.

(B) Additional immunofluorescence images of SGs containing G3BP1 and FXR1 after PF-562271 or defactinib treatment. Cells were treated for the indicated times and stained for SG markers and nuclei. These images correspond to the quantification shown in Fig. 2K.

(C) Immunofluorescence image of U2OS cells treated with low-dose FAK14 for 24 h and stained for G3BP1, eIF4G, F-actin, and nuclei.

Images are representative of experiments independently repeated at least three times with similar results.

Supplementary Fig. S2. Validation of the ROCK–PERK–eIF2α pathway and translational control experiments.

(A) Immunoblot validation of eIF2α kinase knockout MDA-MB-468 cell lines. GCN2, PKR, PERK, and loading controls were analyzed in WT, ΔGCN2, ΔPKR, ΔHRI, and ΔPERK cells.

(B) Immunoblot analysis of sodium arsenite-induced eIF2α phosphorylation in MDA-MB-468 WT and eIF2α kinase-deficient cells.

(C) Immunofluorescence images of FAK14-induced SG formation in U2OS WT and ΔPERK cells. Cells were stained for G3BP1 and eIF4G.

(D) Immunofluorescence images of FAK14-induced SG formation in HAP1 WT and eIF2α S51A cells. Cells were stained for G3BP1 and eIF4G.

(E) Immunofluorescence images of sodium arsenite-induced SG formation in HAP1 WT and eIF2α S51A cells.

(F) Immunofluorescence images showing the effect of Y-27632 on FAK14-induced SG formation in MDA-MB-468 cells. Cells were stained for G3BP1 and eIF4G.

White boxes indicate the regions that were enlarged and used for the corresponding main figure panels. Images are representative of experiments independently repeated at least three times with similar results.

Supplementary Fig. S3. Additional pathway analysis and imaging of SG proteomic datasets.

(A) Pathway analysis of sodium arsenite-selective SG components. Enriched functional categories are shown.

(B) Pathway analysis of SG components shared by sodium arsenite and FAK14 treatment. Shared SG-associated proteins were analyzed for enriched functional categories.

(C) Immunofluorescence images showing CASP4 localization in WT and G3BP1/2 KO cells after FAK14 treatment. Cells were stained for G3BP1, CASP4, and nuclei.

(D) Immunofluorescence images showing CASP4 and eIF4G localization in FAK14-induced SGs.

Images are representative of experiments independently repeated at least three times with similar results.

Supplementary Fig. S4. Basal PTEN and S6 signaling in breast cell lines.

(A) Immunoblot analysis of PTEN, phospho-S6, total S6, and loading controls in MCF10A cells and a panel of breast cancer cell lines. Relevant mutation status is indicated. Basal mTORC1-linked signaling was compared between non-transformed MCF10A cells and breast cancer cell lines.

Immunoblots are representative of experiments independently repeated with similar results.

Supplementary Fig. S5. Validation and supporting data for G3BP-dependent SG assembly, proliferation, viability, and migration.

(A,B) Immunoblot validation of G3BP1 and G3BP2 expression in U2OS and MDA-MB-468 WT and G3BP1/2 KO cells.

(C) Calcein AM live-cell viability assay in MDA-MB-468 WT and G3BP1/2 KO cells treated with FAK14. Live-cell percentages were calculated after background subtraction and normalization to untreated controls.

(D) Immunoblot validation of G3BP1 and G3BP2 expression in G3BP1 KO, G3BP2 KO , G3BP1/2 KO in U2OS cells.

(E–G) Immunofluorescence images and quantification of sodium arsenite-induced SG formation in U2OS WT, G3BP1 KO, G3BP2 KO, and G3BP1/2 KO cells. Cells were stained for eIF4G and nuclei, and SG-positive cells and SG size were quantified.

(H–K) Ki-67 staining images and quantification of proliferating U2OS and MDA-MB-468 WT and G3BP1/2 KO cells. Cells were stained for Ki-67 and nuclei, and the percentage of strongly Ki-67-positive cells was quantified.

(L) Images of U2OS WT and G3BP1/2 KO cells under complete medium and HBSS conditions.

(M) Immunoblot validation of G3BP1 loss in WT and ΔG3BP1 MEFs.

(N) Quantification of Transwell migration in U2OS WT, G3BP1 KO, G3BP2 KO, and G3BP1/2 KO cells. Relative migration was normalized to WT cells.

(O–R) Transwell migration images of U2OS WT and G3BP1/2 KO cells, U2OS G3BP1/2 KO cells reconstituted with EGFP-empty, EGFP-G3BP1 WT, or EGFP-G3BP1-ΔNTF2, and MDA-MB-468 G3BP1/2 KO cells reconstituted with the same constructs. Migrated cells are indicated by arrows where shown.

(S) Immunoblot validation of U2OS cells lacking SG-associated factors, including CSDE1, YB-1, UBAP2L, or FXR-family proteins.

(T) Images from migration assays using U2OS cells deficient in the indicated SG-associated factors.

Data are shown as mean ± SD where quantified. Images and immunoblots are representative of experiments independently repeated at least three times with similar results.

Supplementary Fig. S6. FAZ-3532 inhibits SG assembly and preserves membrane integrity at the indicated doses.

(A) Immunofluorescence images showing the effect of FAZ-3532 on sodium arsenite-induced SG formation in U2OS cells. Cells were treated with vehicle, FAZ-3532, sodium arsenite, or sodium arsenite plus FAZ-3532 and stained for G3BP1 and eIF4G.

(B–D) Quantification of SG formation in U2OS cells treated as in (A). SG-positive cells were quantified in (B), and G3BP1-positive and eIF4G-positive SG numbers per cell were quantified in (C) and (D), respectively.

(E) Immunofluorescence images showing the effect of FAZ-3532 on sodium arsenite-induced SG formation in HeLa cells. Cells were stained for G3BP1 and eIF4G.

(F–H) Quantification of SG formation in U2OS cells treated as in (E). SG-positive cells were quantified in (F), and G3BP1-positive and eIF4G-positive SG numbers per cell were quantified in (G) and (H), respectively.

(I) Ethidium homodimer-1 viability staining in MDA-MB-468 cells treated with increasing concentrations of FAZ-3532 for 2 days. Triton X-100 treatment was used as a positive control for loss of membrane integrity. Cells were stained with Hoechst and EthD-1.

Data are shown as mean ± SD where quantified. Images are representative of experiments independently repeated at least three times with similar results.

Supplementary Figure S1 Related figure 2

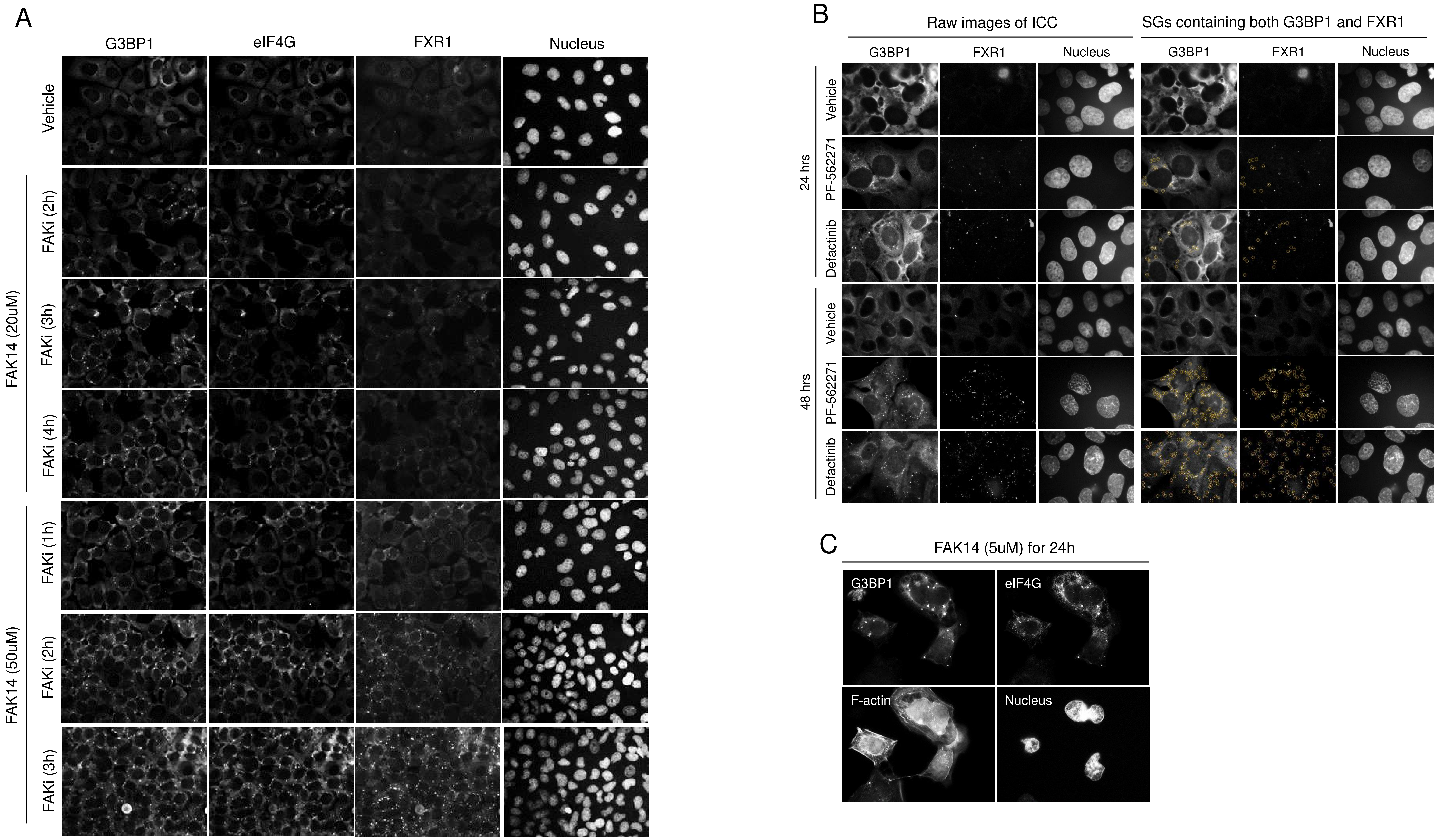

Supplementary Figure S2 Related figure 3

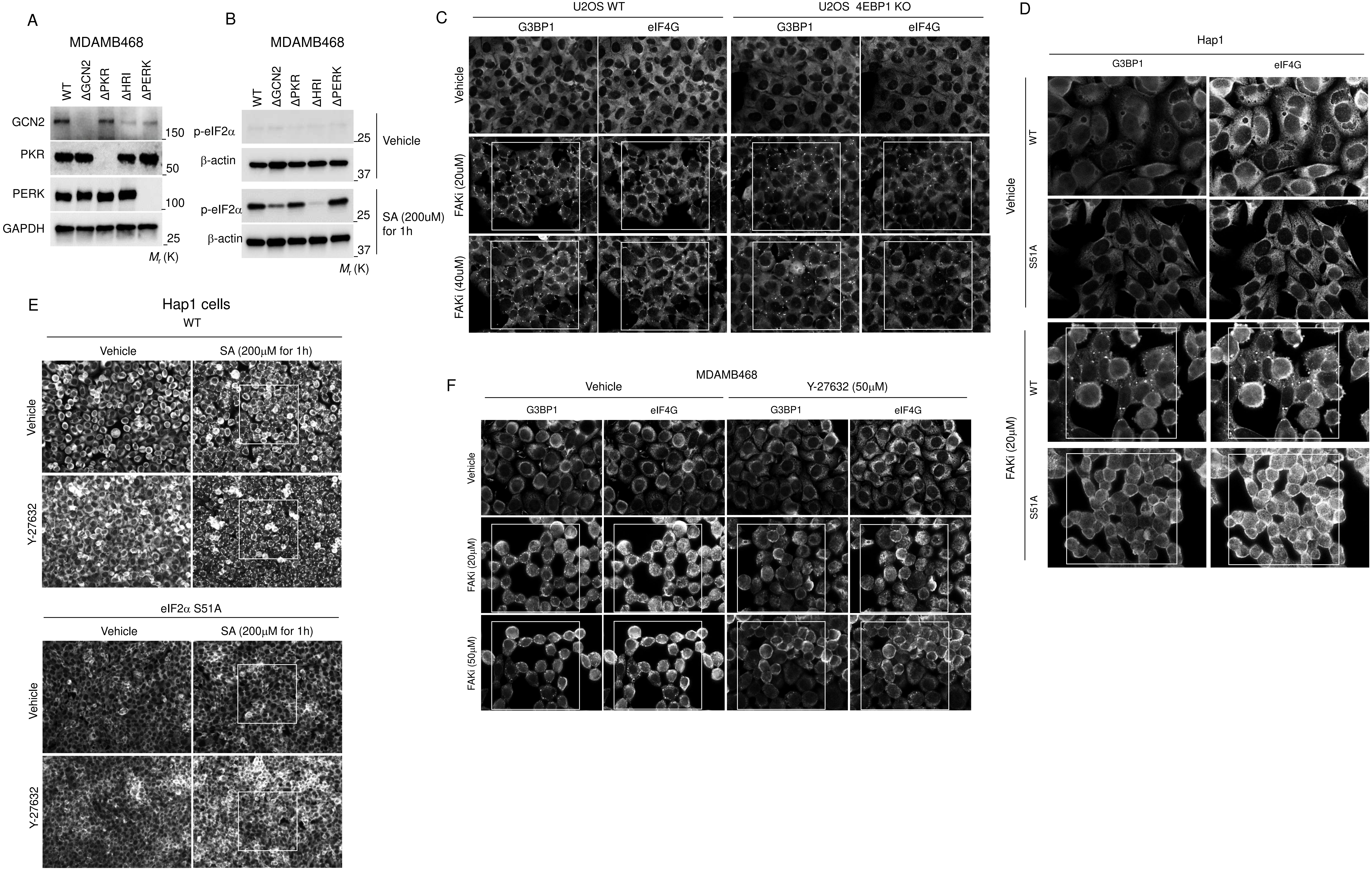

Supplementary Figure S3 Related figure 4

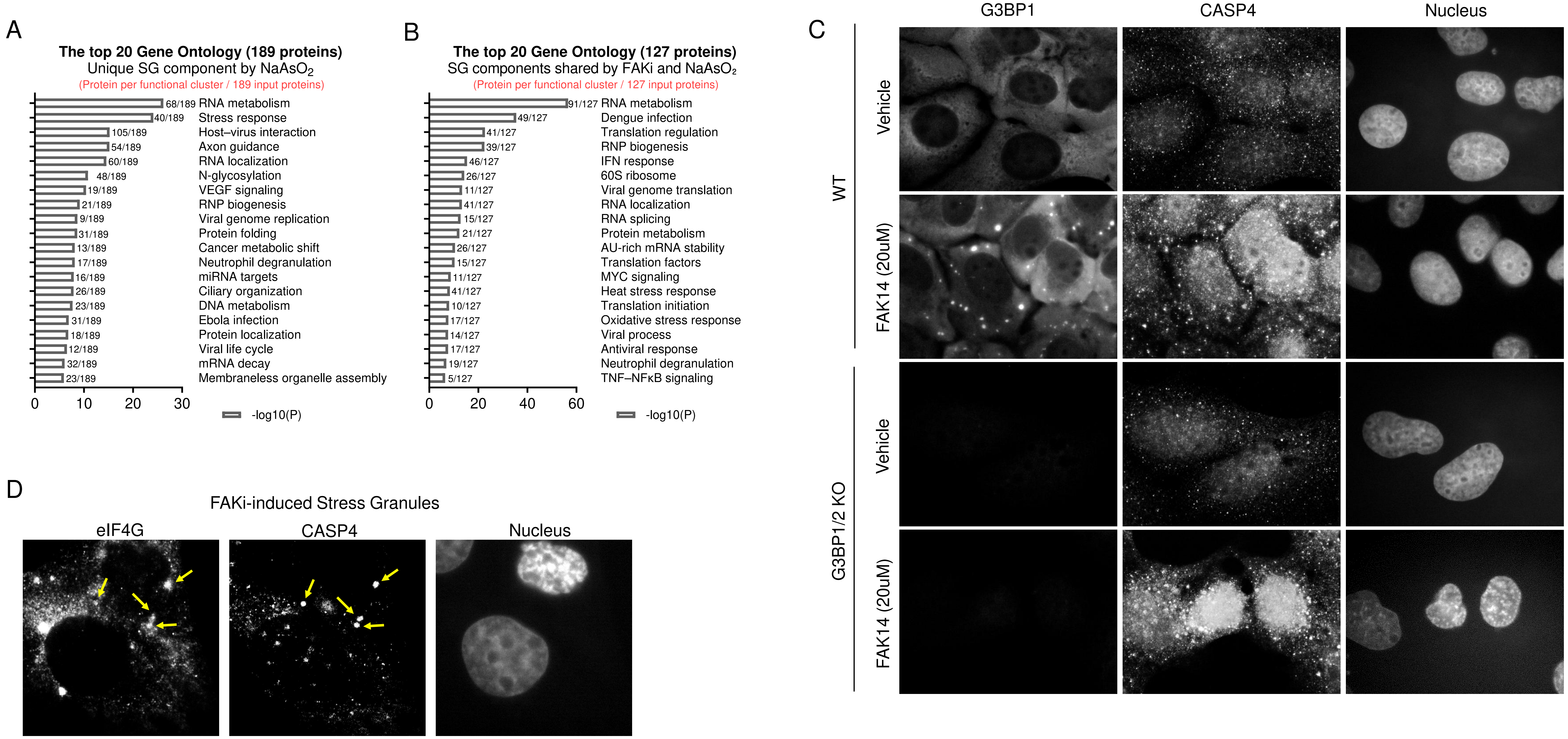

Supplementary Figure S4 Related figure 5

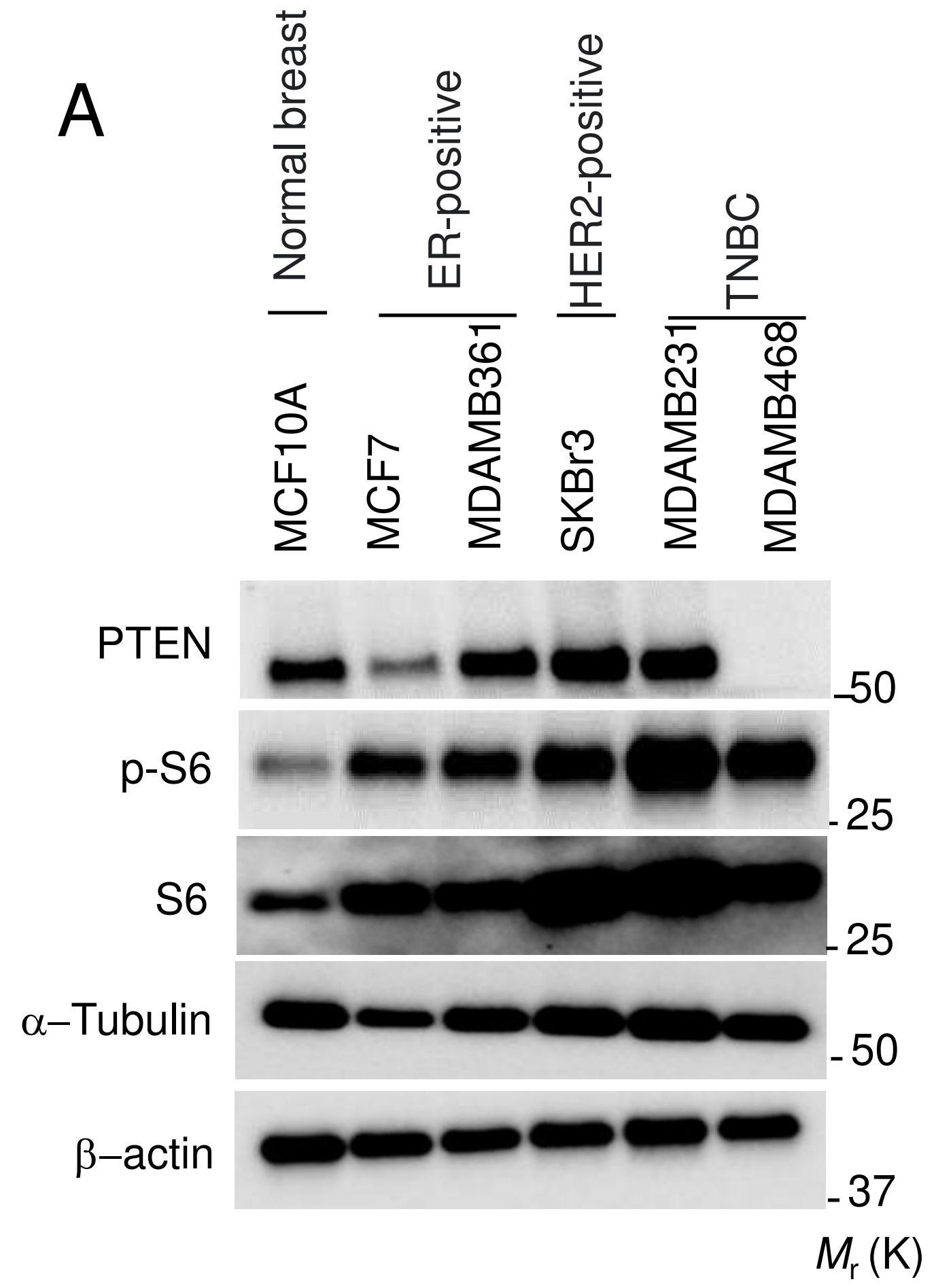

MCF10A : Normal (no mutation)
MCF7: PI3K-active mutation
MDAMB361: PI3K-active mutation
SKBr3: TP53 (mutation)
MDAMB231: KRAS/TP53 (Mutation)
MDAMB468: Loss of PTEN / TP53 (mutation)

Supplementary Figure S5 Related figure 6

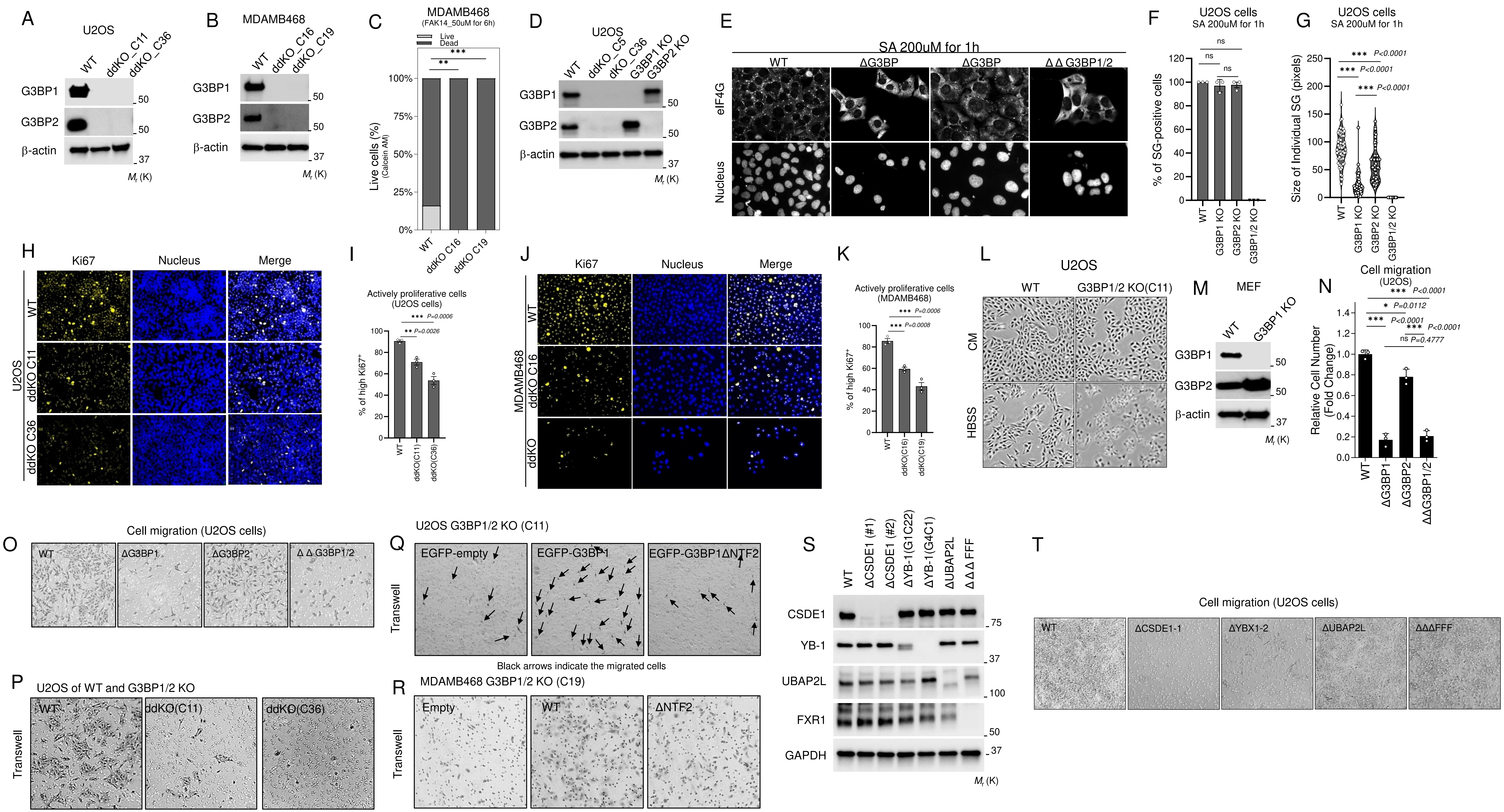

Supplementary Figure S6 Related figure 7

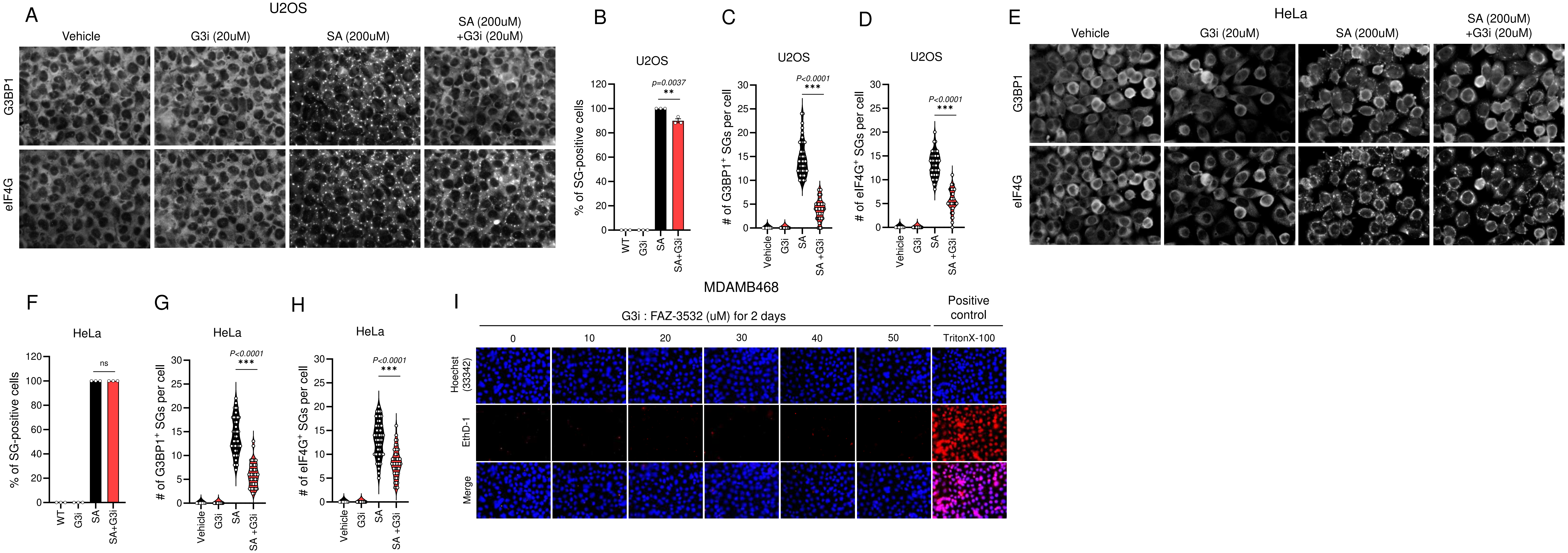

Supplement: Supplementary file 1 [file NIHPPRS10284597V1-supplement-1.pdf]
